# Effect of membrane vesicles produced under different pH conditions on the ability of *Enterococcus faecalis* to tolerate stressful environments and macrophages’ inflammatory response

**DOI:** 10.1128/jb.00377-25

**Published:** 2025-10-16

**Authors:** Poukei Chan, Wenling Huang, Jingheng Liang, Zijian Yuan, Lihong Guo

**Affiliations:** 1 Hospital of Stomatology, Guanghua School of Stomatology, Sun Yat-sen University 623167 , Guangzhou, China; 2 Guangdong Provincial Key Laboratory of Stomatology, Sun Yat-sen University 540446 , Guangzhou, China; University of Southern California, Los Angeles, USA

**Keywords:** *E. faecalis*, membrane vesicles, macrophage, immunoinflammatory responses, transcriptomics

## Abstract

*Enterococcus faecalis* is frequently identified in persistent apical periodontitis lesions and possesses the ability to survive within the alkaline environment of the root canal system after treatment. Previously, we found that the secretion of pro-inflammatory cytokines by macrophages is stimulated by membrane vesicles (MVs) produced by *E. faecalis* at pH levels of 7.0 and 9.0. However, the influence of MVs on the survival of *E. faecalis* under stress conditions and the regulatory impact on macrophage-mediated inflammation require further elucidation. The present study demonstrated that the incorporation of pH 7.0 and 9.0 *E. faecalis* MVs enhances the bacterium’s tolerance to alkaline conditions, hyperosmolarity, oligotrophic environments, and chlorhexidine gluconate. Furthermore, the findings revealed that *E. faecalis* MVs at these pH levels induce M1 polarization of macrophages and promote intracellular ROS production. Transcriptomic analysis and quantitative reverse transcription polymerase chain reaction data indicated that, following treatment of macrophages with MVs under neutral and alkaline conditions, 208 and 302 differentially expressed genes (DEGs), respectively, were identified. These DEGs are predominantly involved in cytokine and cytokine receptor signaling pathways, chemokine signaling pathways, and NOD-like receptor signaling pathways. In summary, the results demonstrate that *E. faecalis* MVs produced under neutral and alkaline conditions contribute to the bacterium’s resistance to extreme environments and play a regulatory role in modulating macrophage inflammatory responses via multiple cytokine signaling pathways.

**IMPORTANCE:**

Persistent apical periodontitis is a prevalent oral disease that may result in tooth loss. *Enterococcus faecalis* is a common pathogen in the root canals of persistent apical periodontitis, exhibiting a remarkable capacity to endure extreme stress conditions. *E. faecalis* has been observed to secrete membrane vesicles, which are associated with bacterial pathogenicity, stress responses, and modulation of host immune regulation. The findings of this study suggest that the release of membrane vesicles by *E. faecalis* under varying pH conditions is correlated with the regulation of macrophage immune-inflammatory responses and the ability to withstand stressful environments. This research provides valuable insights into the pathogenic mechanisms underlying persistent *E. faecalis* infections in persistent apical periodontitis.

## INTRODUCTION

Apical periodontitis (AP) is an oral inflammatory and immune response disease caused by bacterial invasion, resulting in pulpal necrosis and destruction of the alveolar bone in the periapical tissue (1–3). Persistent apical periodontitis (PAP) may occur in 5%–15% of cases following properly performed root canal treatments (RCTs) (4, 5). The persistence of residual microbial biofilms within the apical region of the root canal and adjacent periapical tissues is a principal factor associated with PAP (6, 7). A study of paired root apices from patients with symptomatic PAP identified *Enterococcus* as one of the most prevalent genera (8.10%), present in 47.62% of the periapical lesions (6). *Enterococcus faecalis* is a gram-positive bacterium recognized as one of the most common species isolated in RCT failures, with proportions markedly higher than those observed in primary AP (8–10). It has been noted that *E. faecalis* exhibits a certain tolerance to elevated pH levels and oligotrophic environments and can reproduce in the presence of calcium hydroxide [Ca(OH)_2_], sodium hypochlorite (NaClO), and chlorhexidine gluconate (CHX) (11–15). Moreover, *E. faecalis* virulence factors may promote adherence to the root canal system, thereby complicating eradication even after instrumentation, and potentially exacerbating the inflammatory response of the periapical tissues (16). Therefore, *E. faecalis* should consistently be regarded as a focal point in therapeutic interventions during the treatment or retreatment of root canal infections.

Macrophages play a crucial role in recognizing and eliminating exogenous pathogens in periapical lesions and have been instrumental in the advancement of PAP (17–19). Research indicates that the ratio of macrophage M1 (CD64^+^CD80^+^)/M2 (CD163^+^CD206^+^) in symptomatic periapical lesions is significantly higher compared to asymptomatic stages, with pro-inflammatory cytokines such as IL-1β, IL-6, IL-12, TNF-α, and IL-23 being upregulated (20, 21). An increased proportion of M1 macrophages may contribute to chronic inflammation and exacerbate periapical bone destruction. Consequently, the interaction between pathogens and macrophages remains critical in the progression of PAP. *E. faecalis* has been shown to activate the immune response and stimulate the production of inflammatory cytokines and chemokines (18, 22, 23). Furthermore, Polak et al. (24) have demonstrated that *E. faecalis* infection of macrophages can elevate intracellular reactive oxygen species (ROS) levels by a factor of two within 24 h. A persistent interaction between *E. faecalis*, originating from the root canal, and macrophages within the periapical lesion is likely to occur.

Membrane vesicles (MVs) are produced by cells under physiological and pathological conditions and are secreted extracellularly (25). The generation of MVs is common among both gram-negative and gram-positive oral bacteria and can facilitate inflammation (26). *Porphyromonas gingivalis* outer membrane vesicles (OMVs) have been shown to activate various Toll-like receptors (TLRs), including TLR7, TLR8, TLR9, TLR2, and TLR4, thereby inducing localized chronic inflammation (27). *Fusobacterium nucleatum* OMVs have been demonstrated to promote the transition of M1 macrophages and the release of pro-inflammatory cytokines, thus exacerbating the progression of periodontitis (28). *Staphylococcus aureus* MVs can activate TLR2 signaling in macrophages, resulting in the release of IL-1β and IL-18 (29). Additionally, *S. aureus* MVs also promote the production of IL-6, IL-8, and CCL2 (30). Studies have indicated that *E. faecalis* can also secrete MVs (31–33). Research findings reveal that MVs secreted by *E. faecalis* ATCC 29212 induce periapical bone resorption and possess the capacity to polarize M1 macrophages (31). While *E. faecalis* MVs have been associated with inflammation, further investigation is necessary to elucidate the interaction between MVs released by *E. faecalis* in different pH environments and macrophages.

In our preceding study (34, 35), an alkaline environment with a pH of 9.0 did not influence the production of *E. faecalis* MVs. Proteomic and metabolomic analyses revealed that the expression levels of ATP synthase and stress response proteins increased, as well as the pathways involved in lysine degradation and tryptophan metabolism, which were significantly enriched in *E. faecalis* MVs derived under pH 9.0 conditions. Furthermore, it was determined that *E. faecalis* MVs produced at pH 7.0 and 9.0 exhibited a dose-dependent inhibition of the activity of the differentiated THP-1 cells (dTHP-1 cells), alongside a promotion of cytokine secretion—including IL-1β, IL-6, TNF-α, IL-1ra, and TGF-β—by macrophages, and a significant increase in the IL-1β/IL-1ra ratio. To facilitate a comprehensive investigation into the impact of *E. faecalis*-produced MVs under different pH conditions on bacterial resilience to extreme environments and macrophage response, this study examined the effect of *E. faecalis* MVs on the survival capacity of *E. faecalis* in stressful conditions. Additionally, this study investigated the regulatory inflammatory impact of *E. faecalis* MVs derived from cultures at different pH levels on macrophage polarization and ROS generation, as well as their effects on the macrophage transcriptome, through *in vitro* experiments. This research enhances our understanding of the virulence factors of *E. faecalis* and offers new insights into the pathogenic mechanisms involved in persistent periapical infections associated with *E. faecalis*.

## RESULTS

### *E. faecalis* MVs produced under different pH conditions enhance *E. faecalis*’s tolerance to environmental stress

**Fig 1:**
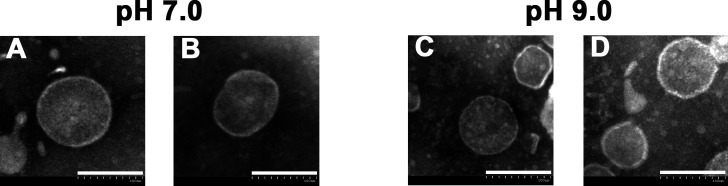
Transmission electron microscopy images of *E. faecalis* MVs isolated from bacterial culture media at pH 7.0 (**A and B**) and pH 9.0 (**C and D**), magnified at ×40,000 (scale bar: 100 nm).

Transmission electron microscopy (TEM) demonstrated that *E. faecalis* produced characteristic spherical vesicles at pH 7.0 and 9.0, displaying a lipid bilayer membrane structure with an approximate diameter of 100 nm (Fig. 1). Our preliminary investigations have determined that pH 7.0 MVs exhibited a particle size of 139.9 ± 67.0 nm. In comparison, pH 9.0 MVs demonstrated a particle size of 129.5 ± 75.9 nm, indicating that an alkaline environment promotes the formation of smaller *E. faecalis* vesicles (*P* < 0.05) (35). Consequently, the concentrations and quantities of pH 7.0 and 9.0 MVs are not equal. It is imperative to emphasize that all concentrations reported in this study have been normalized based on protein content.

**Fig 2:**
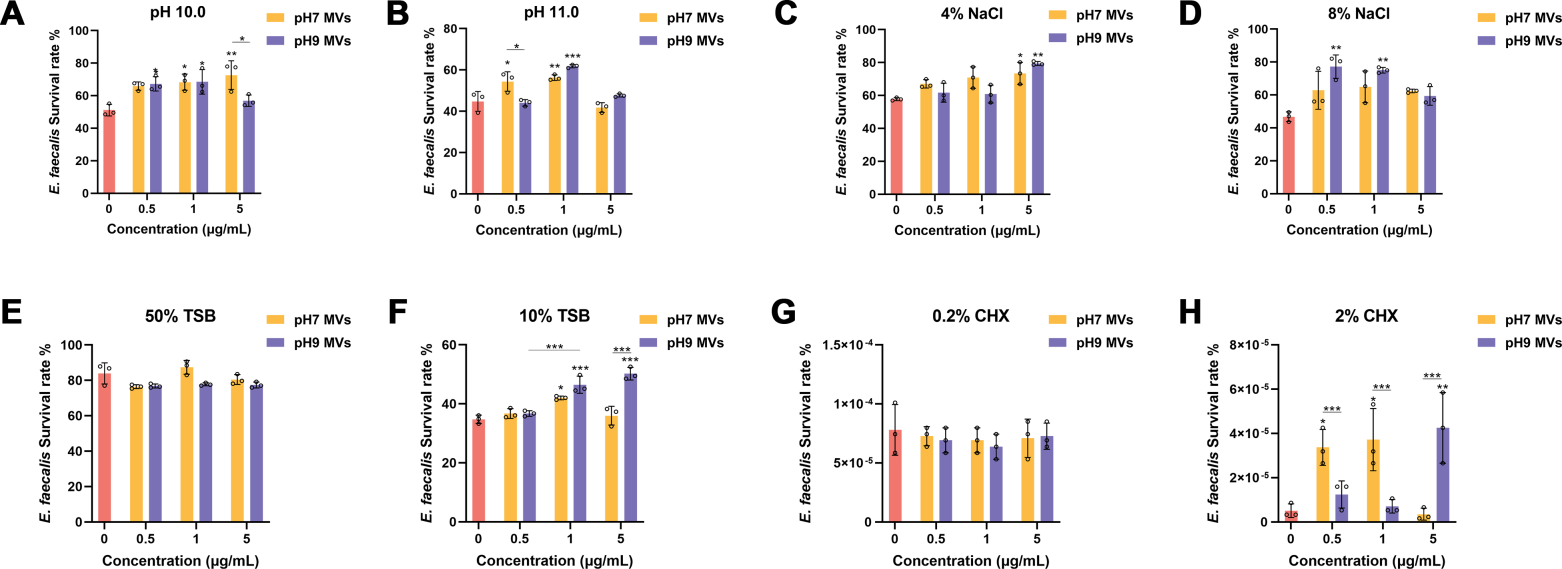
The survival of *E. faecalis* under various environmental stresses following the incorporation of *E. faecalis* MVs produced under differing pH conditions. (**A and B**) Survival of *E. faecalis* in alkaline environments. (**C and D**) Survival of *E. faecalis* in hyperosmolar conditions. (**E and F**) Survival of *E. faecalis* in oligotrophic environments. (**G and H**) Survival of *E. faecalis* in culture medium supplemented with CHX for 1 min. The assays were conducted in triplicate. Statistical analysis was carried out using one-way analysis of variance for multiple comparisons, supplemented by Bonferroni’s post-correction. Error bars denote the mean with SD. *P < 0.05, **P < 0.01, and ***P < 0.001.

Under conditions of pH 10.0, there was a notable increase in the survival rate of *E. faecalis* within the groups treated with 1.0 and 5.0 µg/mL of pH 7.0 *E. faecalis* MVs, as well as 0.5 and 1.0 µg/mL of pH 9.0 *E. faecalis* MVs (*P < 0.05). No statistically significant difference was identified between the two groups receiving 1.0 µg/mL (*P* > 0.05). Within the pH 7.0 and pH 9.0 groups, no concentration-dependent effect was observed (*P* > 0.05) (Fig. 2A). At pH 11.0, the inclusion of 1.0 µg/mL of *E. faecalis* MVs at pH 7.0 and 9.0 resulted in a statistically significant increase in the survival rate of *E. faecalis* (**P < 0.01 and ***P < 0.001). However, no significant difference was found between the two groups (*P* > 0.05) (Fig. 2B).

The incorporation of pH 7.0 and pH 9.0 *E. faecalis* MVs at a concentration of 5.0 µg/mL led to a notable enhancement in the survival capacity of *E. faecalis* within a 4% NaCl medium (*P < 0.05 and **P < 0.01) (Fig. 2C). Conversely, in the 8% NaCl medium, survival rates demonstrated a significant increase exclusively in groups supplemented with 0.5 and 1.0 µg/mL pH 9.0 *E. faecalis* MVs (**P < 0.01) (Fig. 2D).

The addition of *E. faecalis* MVs into a 50% tryptic soy broth (TSB) medium did not produce a statistically significant influence on the survival rate of *E. faecalis* (*P* > 0.05) (Fig. 2E). Conversely, in a 10% TSB medium, the addition of 1.0 µg/mL of *E. faecalis* MVs at pH 7.0 and 9.0 led to a significant augmentation in the survival rate of *E. faecalis* (*P < 0.05 and ***P < 0.001). Notably, the group treated with pH 9.0 MVs demonstrated effects that were dependent on concentration (Fig. 2F).

Following treatment with 0.2% CHX for 1 min, no statistically significant differences in survival rates were observed between the two groups treated with *E. faecalis* MVs and the control group (*P* > 0.05) (Fig. 2G). Subsequently, after treatment with 2% CHX for 1 min, the addition of 0.5 and 1.0 µg/mL of pH 7.0 *E. faecalis* MVs, as well as 5.0 µg/mL of pH 9.0 *E. faecalis* MVs, resulted in a significant increase in the survival rate of *E. faecalis* (***P < 0.001 and **P < 0.01) (Fig. 2H). Furthermore, *E. faecalis* was unable to survive in any group following 3 min of exposure to 0.2% and 2% CHX.

### *E. faecalis* MVs produced under neutral and alkaline environments induce macrophage M1 polarization

**Fig 3:**
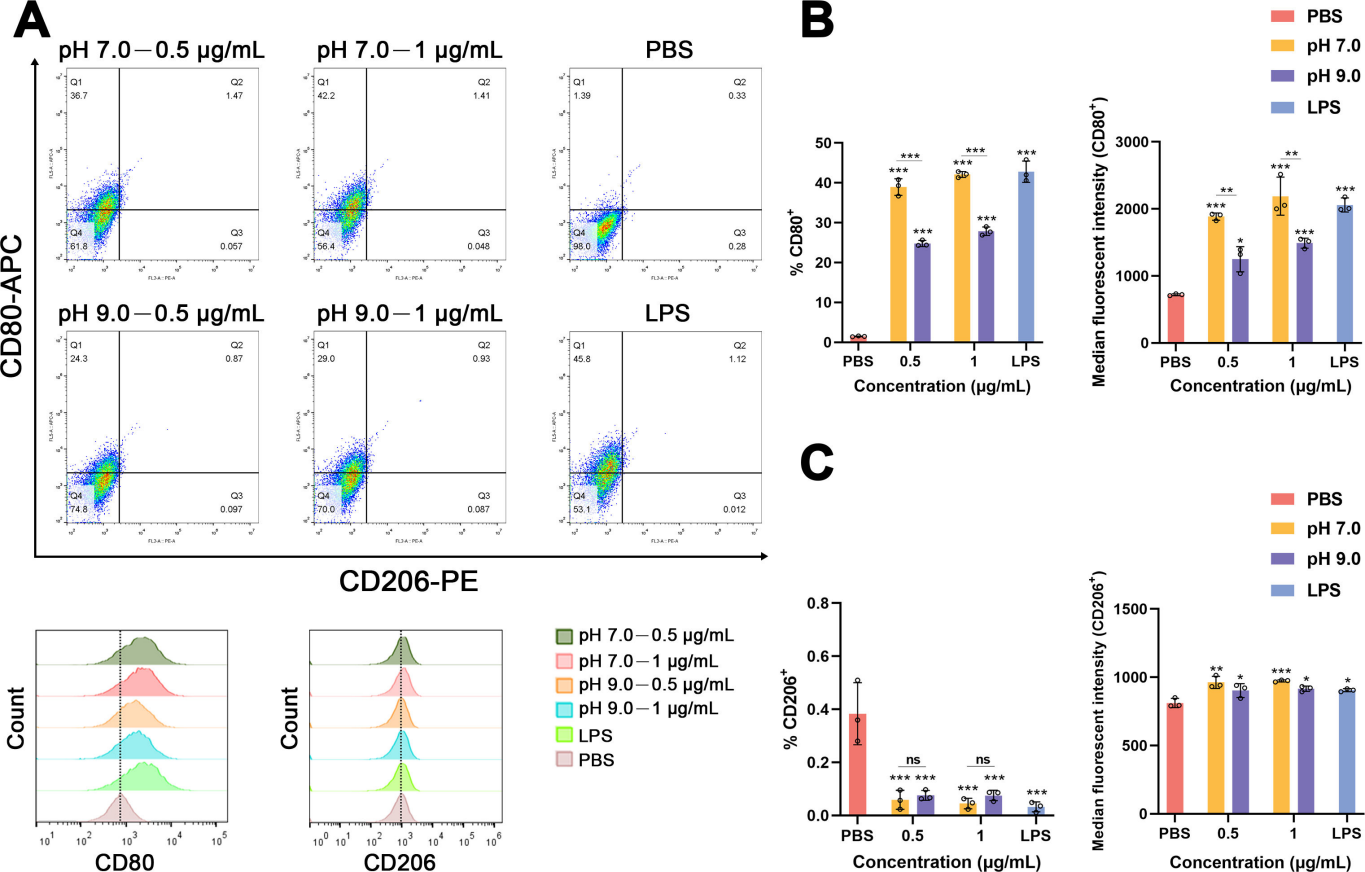
Flow cytometry analysis of dTHP-1 cells treated with *E. faecalis* MVs produced under varying pH conditions, as well as treated with phosphate-buffered saline (PBS) and lipopolysaccharide (LPS) (0.5 µg/mL), stained with CD80-APC and CD206-PE. (**A**) Dot plots and histograms obtained through flow cytometry of dTHP-1 cells. (**B**) Percentage of CD80^+^ macrophages and median fluorescent intensity of CD80-APC. (**C**) The proportion of CD206^+^ macrophages and the median fluorescent intensity of CD206-PE. The experiment was conducted in triplicate. Statistical analysis was performed utilizing one-way analysis of variance for multiple comparisons, accompanied by Bonferroni’s post-correction. The data are presented as mean with SD. *P < 0.05, **P < 0.01, ***P < 0.001, and ns, not significant.

To illustrate the different macrophage subpopulations, the percentages of M1 macrophage marker CD80^+^CD206^-^ and M2 macrophage marker CD206^+^CD80^-^ on treated dTHP-1 cells were examined. The findings showed that after 24 h of *E. faecalis* MVs administration, the proportion of CD80^+^CD206^-^ cells significantly increased across all treatment groups (***P < 0.001), suggesting that both pH 7.0 and pH 9.0 MVs markedly promoted macrophage polarization toward the M1 phenotype (Fig. 3). The analysis of the proportion of CD80^+^ cells and the median fluorescent intensity indicates that the impact of *E. faecalis* MVs produced under neutral conditions was significantly more substantial than that under alkaline conditions (***P < 0.001). It was also observed that there was no significant difference in induction effects between varying concentrations within the two treatment groups (*P* > 0.05) (Fig. 3B). Moreover, the proportion of cells expressing CD206^+^CD80^-^ was significantly reduced in all treatment groups relative to the negative control (***P < 0.001). Although the median fluorescent intensity demonstrates a statistically significant increase relative to the control group (*P < 0.05, **P < 0.01, and ***P < 0.001), no significant differences were identified between the experimental groups (*P* > 0.05) (Fig. 3C).

### *E. faecalis* MVs produced under different pH conditions promote the production of ROS in macrophages

**Fig 4:**
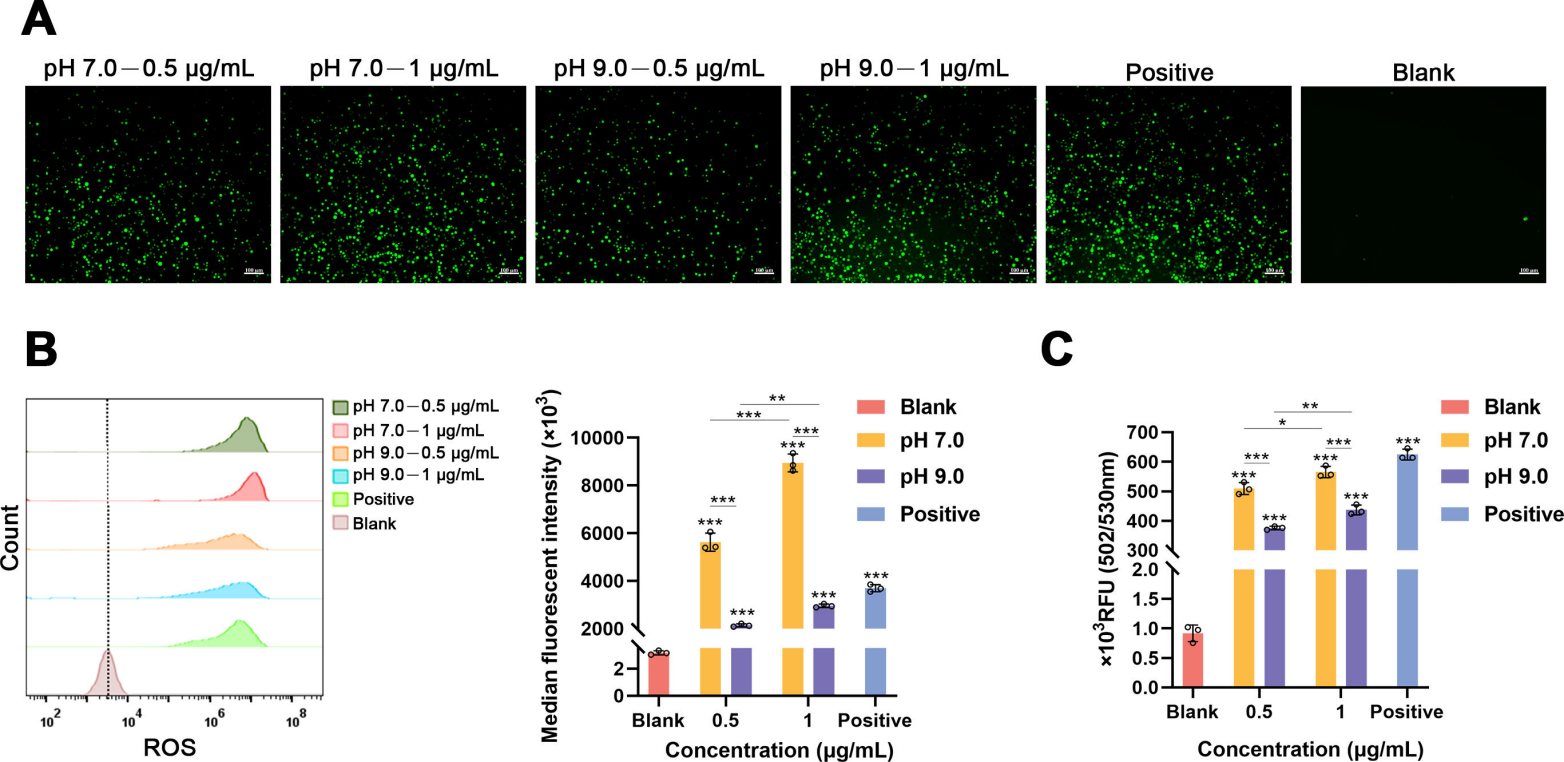
Detection of ROS production using the fluorescent probe DCFH-DA. Macrophages exposed to H_2_O_2_ at a concentration of 48 µM were utilized as a positive control. (**A**) Representative fluorescence microscopy images depicting ROS in green (scale bar: 100 µm). (**B**) Median fluorescent intensity based on the flow cytometry of intracellular ROS levels. (**C**) Optical density measurements were obtained via a fluorescence plate reader. The assays were conducted in triplicate. Statistical analysis was performed using one-way analysis of variance for multiple comparisons, accompanied by Bonferroni’s post-correction. Error bars denote the mean with SD. *P < 0.05, **P < 0.01, and ***P < 0.001. RFU, relative fluorescence unit.

The detection of intracellular ROS production in macrophages following treatment with the 2′7′-dichlorofluorescein diacetate (DCFH-DA) fluorescent probe was conducted. Fluorescence microscopy revealed the presence of ROS, manifested as a green signal (Fig. 4A). The analysis of median fluorescent intensity and relative fluorescence unit demonstrated that MVs derived from *E. faecalis* under both pH conditions could induce ROS production in macrophages (***P < 0.001). Moreover, the capacity of *E. faecalis* MVs in a neutral environment to stimulate ROS production was markedly greater than that in an alkaline environment (***P < 0.001). Additionally, the ability of *E. faecalis* MVs at pH 7.0 and 9.0 to induce ROS production increased significantly with higher concentrations (*P < 0.05, **P < 0.01, and ***P < 0.001) (Fig. 4B and C).

### The effect of *E. faecalis* MVs produced in neutral and alkaline pH environments on the macrophage transcriptome

**Fig 5:**
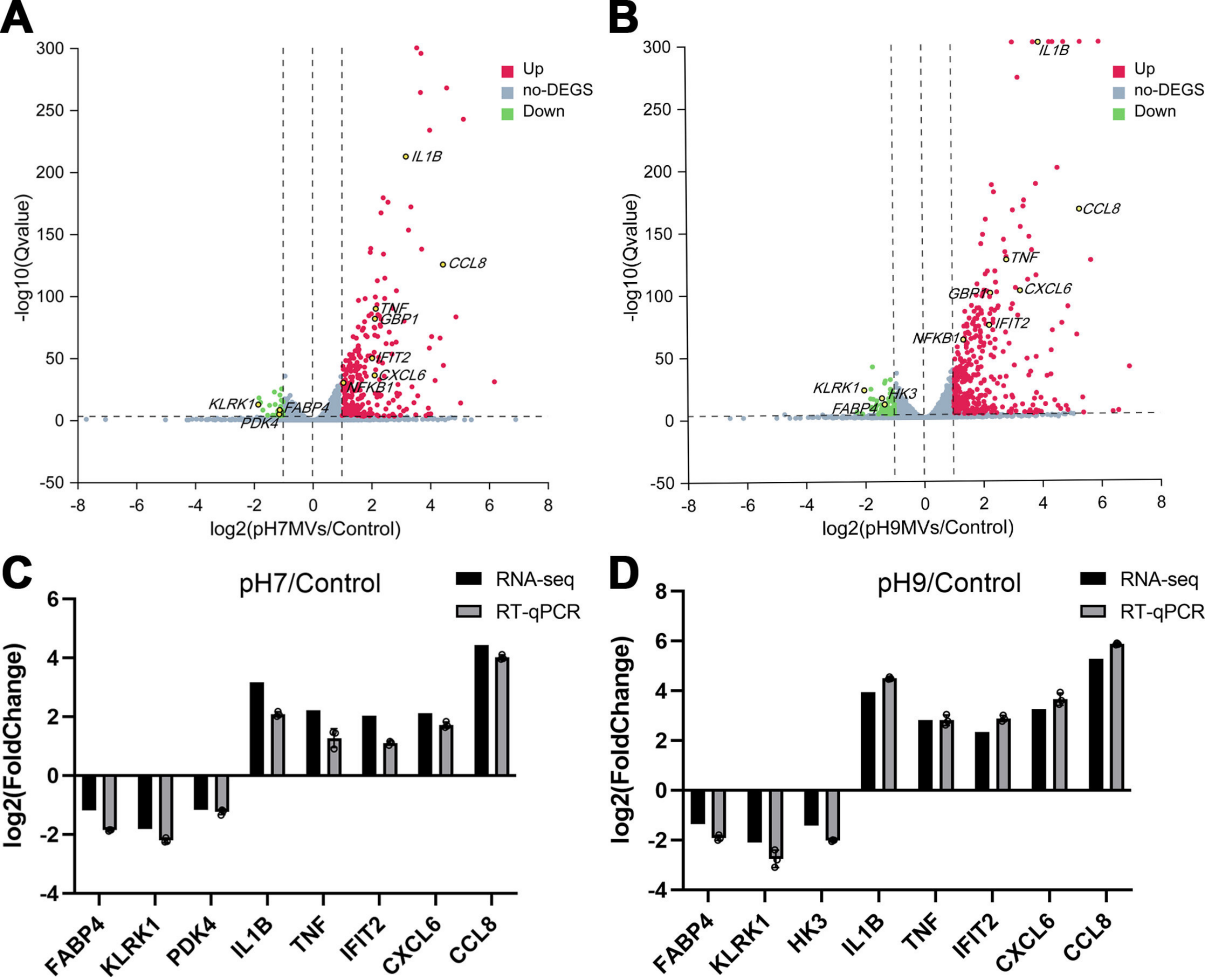
DEGs in macrophages treated with *E. faecalis* MVs produced under varying pH conditions in comparison to the control group. (**A and B**) Volcano plots illustrate DEGs. Each plot represents a gene. DEGs with a Log2 fold change ≥1 are accentuated in red, signifying upregulated genes, while DEGs with a Log2 fold change ≤ −1 are accentuated in green, indicating downregulated genes (false discovery rate < 0.001). Gray represents no significant difference. (**C and D**) Validation of DEGs identified by RNA sequencing with confirmation by RT-qPCR. The experiment was conducted in triplicate. Data are presented as mean with SD.

Compared to the control group, a significant number of differentially expressed genes (DEGs) were identified in macrophages treated with *E. faecalis* MVs (|Log2 fold change| ≥ 1 and false discovery rate < 0.001). Specifically, macrophages exposed to pH 7.0 *E. faecalis* MVs exhibited differential expression of 208 genes, comprising 188 upregulated and 20 downregulated genes. Comparing the results of those treated with pH 9.0 *E. faecalis* MVs showed differential expression of 302 genes, with 260 upregulated and 42 downregulated (Fig. 5A and B). Comprehensive details regarding the DEGs are provided in Tables S1 and S2. The expression of DEGs, which are associated with inflammatory pathways identified through preliminary experimental results, was further validated using quantitative reverse transcription polymerase chain reaction (RT-qPCR). This validation demonstrated a high level of concordance with the RNA sequencing (RNA-seq) outcomes. A strong correlation was observed between the two data sets, as indicated by Pearson correlation coefficients of 0.9789 and 0.9970, respectively (Fig. 5C and D). For further information concerning the DEGs between the pH 9.0 *E. faecalis* MV group and the pH 7.0 *E. faecalis* MV group (|Log2 fold change| ≥ 0.585 and false discovery rate < 0.001), please refer supplemental materials (Table S3; Fig. S3 to S5).

### The impact of *E. faecalis* MVs formed under neutral conditions on macrophages

**Fig 6:**
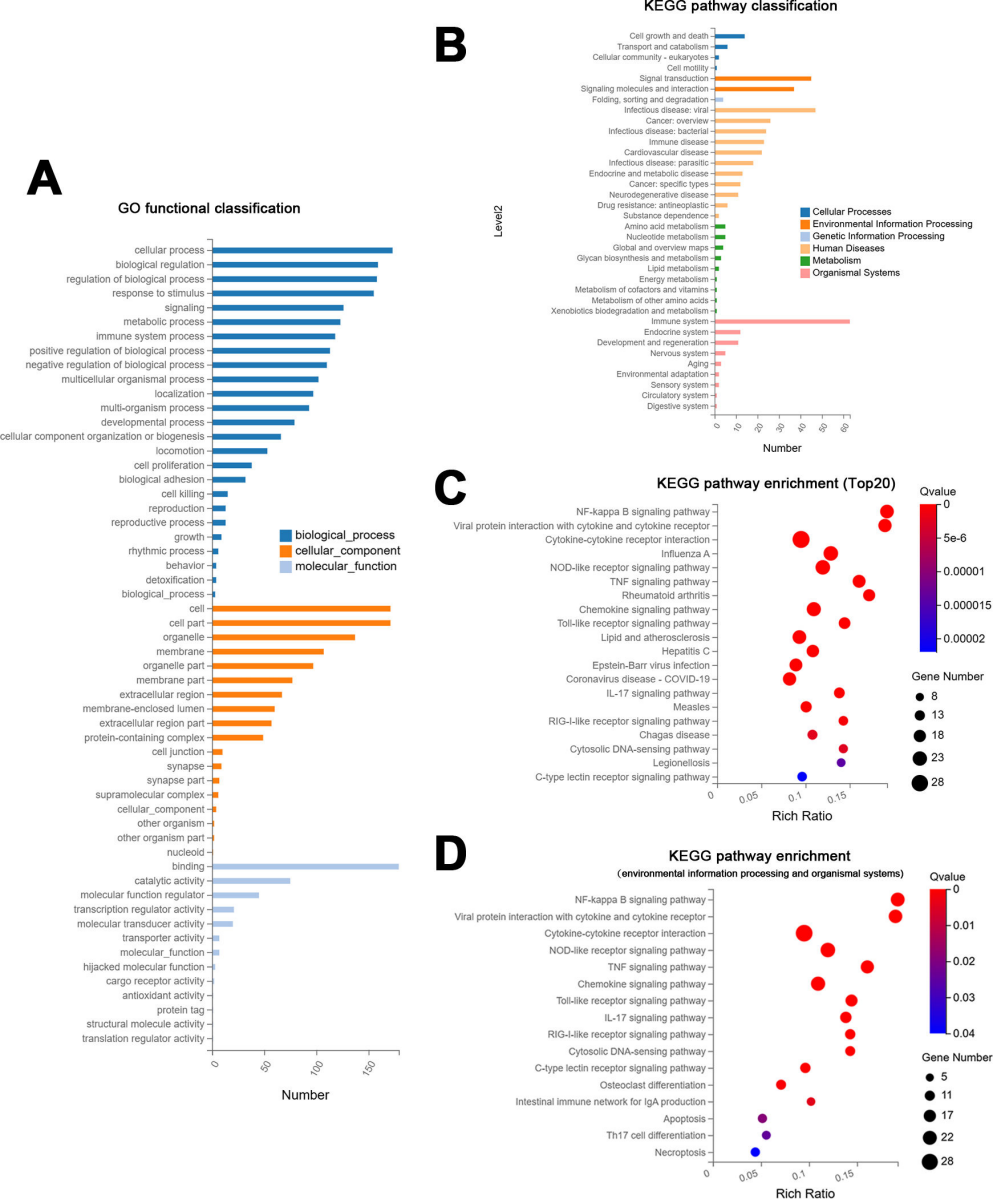
Analysis of DEGs in macrophages subjected to pH 7.0 *E. faecalis* MVs. (**A**) GO functional classifications. (**B**) Significantly enriched KEGG pathway classifications. (**C**) Bubble chart illustrating the top 20 KEGG pathways enriched in the analysis. (**D**) Bubble chart illustrates KEGG pathway enrichment related to environmental information processing and organismal systems.

In GO analysis, the DEGs in macrophages treated with pH 7.0 *E. faecalis* MVs were predominantly enriched in biological processes, notably those related to cellular processes, biological regulation, response to stimulus, signaling, metabolic process, and immune system process. Furthermore, it was observed that the DEGs were also significantly enriched in various cellular components and molecular functions (Fig. 6A).

Following KEGG pathway analysis, DEGs in macrophages exposed to pH 7.0 *E. faecalis* MVs were found to be enriched across multiple pathways, with a significantly higher number of DEGs associated with the immune system. Moreover, DEGs demonstrated substantial enrichment in pathways linked to human diseases, environmental information processing, and cellular processes (Fig. 6B). As illustrated in the KEGG pathway enrichment bubble chart, Fig. 6C demonstrates that the majority of the top 20 enriched pathways, as determined by the false discovery rate, are associated with inflammatory responses. Figure 6D illustrates all significantly enriched KEGG pathways linked to environmental information processing and organismal systems. These enriched pathways included the NF-κB signaling pathway, viral protein interaction with cytokine and cytokine receptor, cytokine-cytokine receptor interaction, NOD-like receptor signaling pathway, TNF signaling pathway, chemokine signaling pathway, Toll-like receptor signaling pathway, IL-17 signaling pathway, and C-type lectin receptor signaling pathway. Additionally, the DEGs were markedly enriched in osteoclast differentiation, apoptosis, Th17 cell differentiation, and necroptosis.

**Fig 7:**
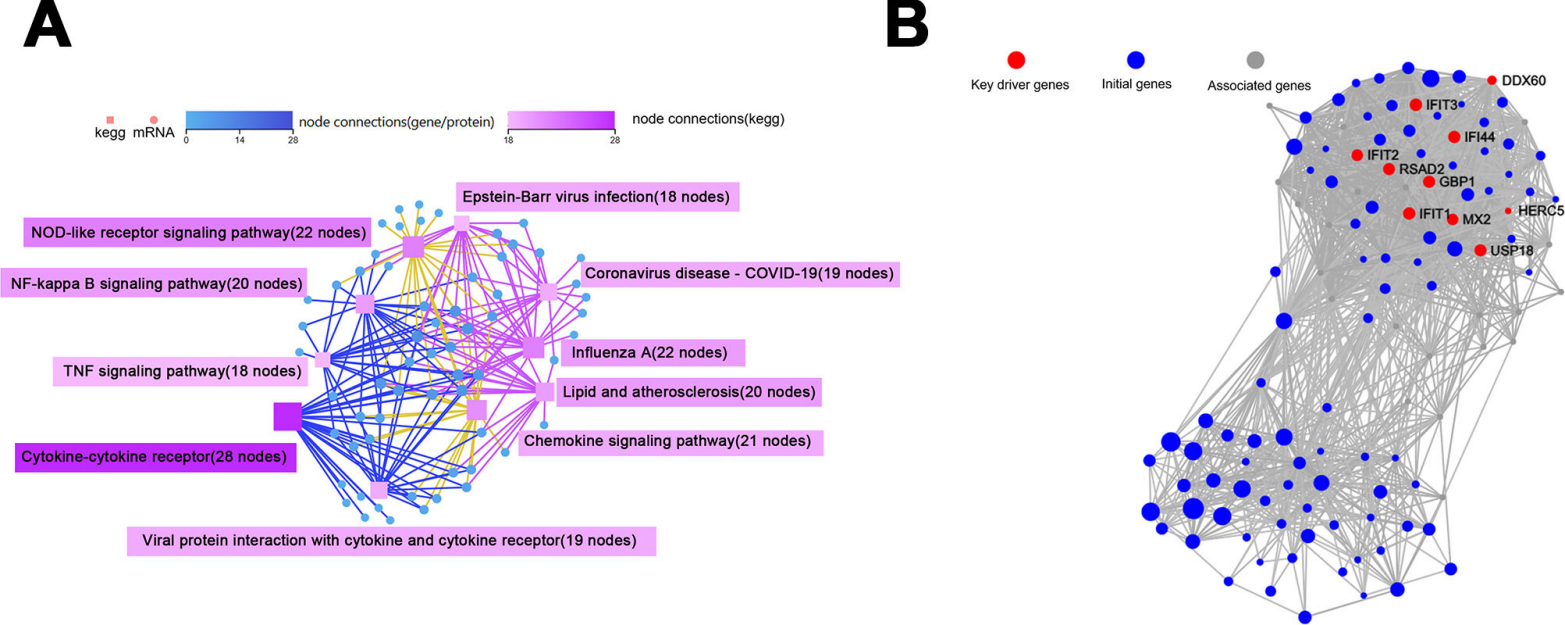
Network interaction analysis of KEGG pathways (**A**) and key driver analysis for protein-protein interactions (**B**), both of which were performed on the DEGs identified in macrophages subjected to pH 7.0 *E. faecalis* MVs.

In the KEGG pathway network-interaction analysis, the connections between the top 10 pathways with the highest number of DEGs were identified. The results demonstrated that the cytokine-cytokine receptor, NOD-like receptor signaling pathway, and chemokine signaling pathway exhibited a greater number of node connections, suggesting that they possess more profound interconnections with other DEGs and enriched pathways (Fig. 7A).

Subsequent analysis of the network interactions of key DEGs was conducted using the protein-protein interaction (PPI) network (Fig. S1). Based on the findings from the PPI analysis, key driver analysis (KDA) was performed to identify the principal driver genes and initial genes (Fig. 7B). The results indicated that the key driver genes were all located within the same cluster, including *GBP1*, *IFIT1*, *IFIT2*, *IFIT3*, *IFI44*, *DDX60*, *MX2*, *HERC5*, *RSAD2*, and *USP18*. Most of these genes are associated with immune responses. The study demonstrates that *GBP1* is associated with the NOD-like receptor signaling pathway, utilizing an extended conformation to interact with bacterial membranes and initiate the innate immune response to infection (36). Additionally, studies have shown that overexpression of *IFIT1* significantly upregulates the expression of inflammatory cytokines in LPS-induced human umbilical vein endothelial cells (37, 38). *IFIT2* contributes to the positive regulation of LPS-induced TNF-α secretion (39). *IFIT3*, *IFI44*, and *DDX60* are interferon-stimulated genes (ISGs) (40–42). Furthermore, studies have demonstrated that *MX2* can activate the NOD-like receptor signaling pathway and promote neutrophil infiltration (43). It has been observed that *HERC5* enhances the activation of NLRP3 inflammasomes (44). Research also indicates that *RSAD2* mRNA and protein expression are markedly increased in THP-1 polarized M1 macrophages (45). *USP18* has been identified to play a regulatory role in interferon response (46).

### Effects of *E. faecalis* MVs produced in an alkaline environment on macrophages

**Fig 8:**
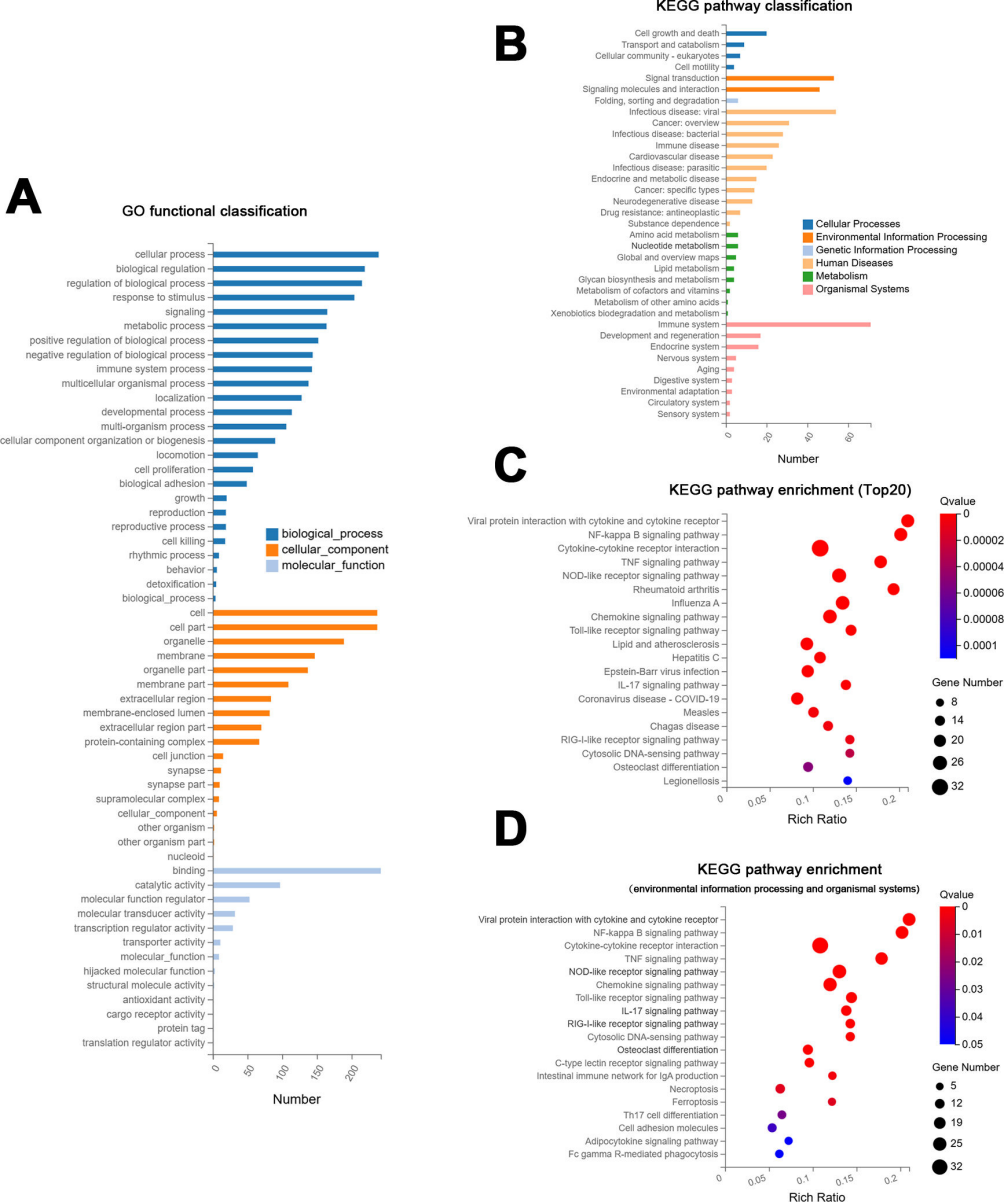
DEGs analysis in macrophages exposed to pH 9.0 *E. faecalis* MVs. (**A**) GO functional classification. (**B**) KEGG pathway classification. (**C**) Bubble chart illustrating the top 20 KEGG pathways in terms of enrichment. (**D**) Bubble chart of KEGG pathway enrichment associated with environmental information processing and organismal systems.

Analogous to the analysis results of DEGs identified following macrophage treatment with neutral environment *E. faecalis* MVs, GO analysis revealed that DEGs in macrophages exposed to pH 9.0 *E. faecalis* MVs were enriched across biological processes, cellular components, and molecular functions, with biological processes including cellular processes, biological regulation, regulation of biological processes, response to stimulus, signaling, and metabolic processes (Fig. 8A).

KEGG pathways were enriched in the organismal systems pathway, human diseases, and environmental information processing pathways (Fig. 8B). As indicated by the KEGG pathway enrichment bubble chart, it is noteworthy that the top five enriched pathways are all related to inflammatory responses. The results of the analysis are similar to the effects caused by *E. faecalis* MVs produced in an environment with a pH of 7.0. Figure 8C depicts the top 20 enriched KEGG pathways as identified by false discovery rate, whereas Fig. 8D displays all significantly enriched KEGG pathways related to environmental information processing and organismal systems. The top 20 enriched pathways comprise interactions between viral protein interaction with cytokine and cytokine receptor, NF-κB signaling pathways, cytokine-cytokine receptor interaction, TNF signaling pathway, NOD-like receptor signaling pathway, chemokine signaling pathway, Toll-like receptor signaling pathway, IL-17 signaling pathway, and osteoclast differentiation. Among them, the osteoclast differentiation pathway was found to be significantly enriched, with a higher number of related DEGs than in a neutral environment. Furthermore, DEGs have been demonstrated to be implicated in the C-type lectin receptor signaling pathway, necroptosis, ferroptosis, Th17 cell differentiation, adipocytokine signaling pathway, and Fc gamma R-mediated phagocytosis.

**Fig 9:**
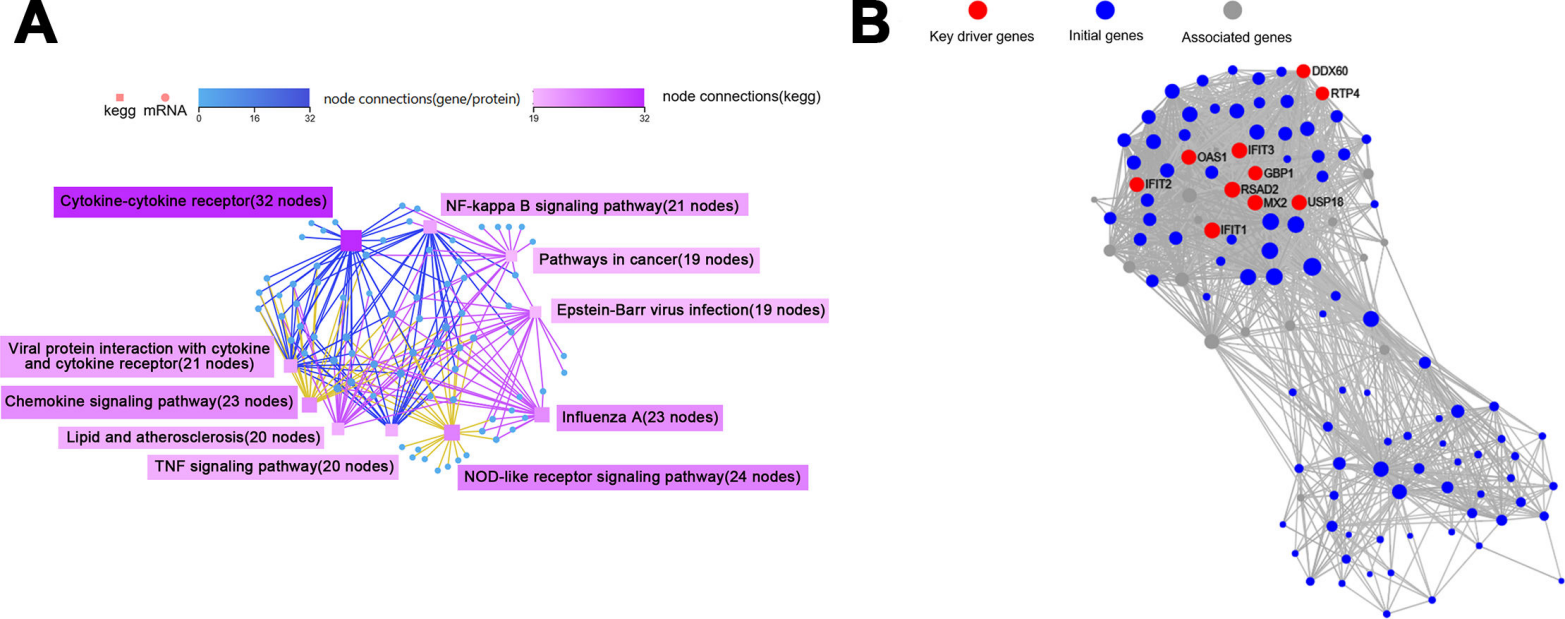
KEGG pathway network interaction analysis (**A**) and a key driver analysis of protein-protein interactions (**B**) were performed on DEGs in macrophages exposed to pH 9.0 *E. faecalis* MVs.

The subsequent analysis will investigate the associations between the top 10 KEGG pathway networks with the highest number of DEGs. The results indicate that, similar to the neutral environment, the cytokine-cytokine receptor, NOD-like receptor signaling pathway, and chemokine signaling pathway display increased node connections, comprising 32, 24, and 23 nodes, respectively (Fig. 9A).

The utilization of PPI was instrumental in facilitating a more comprehensive analysis of the network interactions among DEGs (Fig. S2). Additionally, this methodology enabled the identification of key driver genes through KDA (Fig. 9B). The findings indicated that the key driver genes shared significant similarities with the DEGs analyzed under the influence of pH 7.0 *E. faecalis* MVs, including *GBP1*, *DDX60*, *IFIT1*, *IFIT2*, *IFIT3*, *MX2*, *RSAD2*, and *USP18*. Furthermore, *OAS1* and *RTP4* were also identified in the results. It has been established that the presence of full-length *OAS1* within THP-1 cells enhances their resistance to bacterial infection (47). *OAS1* was also associated with the NOD-like receptor signaling pathway. Additionally, *RTP4* has been characterized as an ISG (48).

### *E. faecalis* MVs produced under varying pH environments upregulate the expression of genes related to the NOD-like receptor signaling pathway and pyroptosis in macrophages

**Fig 10:**
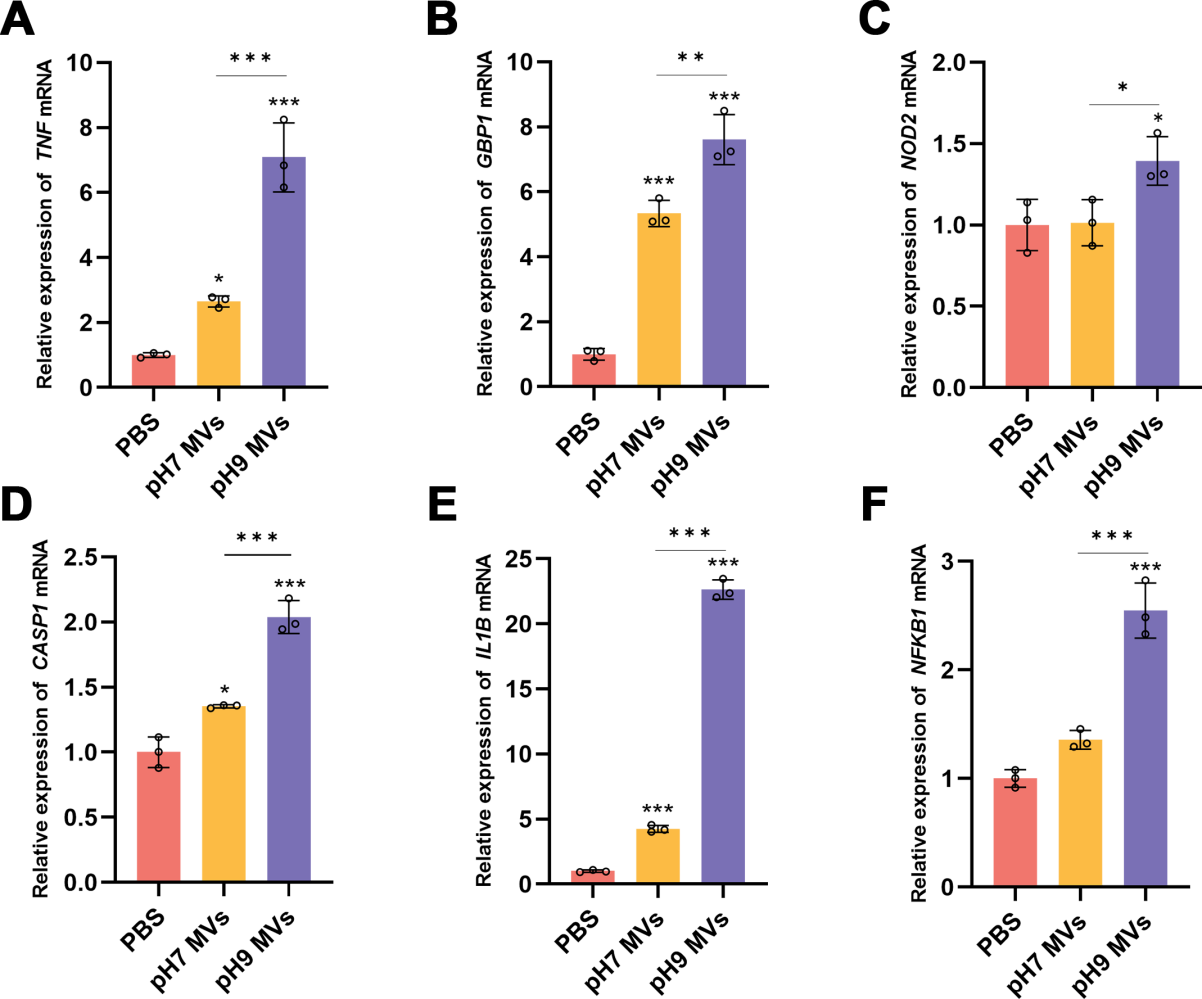
Validation of RT-qPCR results regarding the impact of *E. faecalis* MVs produced under varying pH conditions on macrophage gene expression associated with the NOD-like receptor signaling pathways (**A–C**) and pyroptosis (**D–F**). The assay was conducted in triplicate. Statistical analysis was carried out using one-way analysis of variance for multiple comparisons, with Bonferroni’s *post hoc* correction applied. Error bars denote the mean with SD. *P < 0.05, **P < 0.01, and ***P < 0.001.

The results from the KDA indicate a correlation between several key genes and the NOD-like receptor signaling pathway. RT-qPCR analysis demonstrated that *TNF* and *GBP1*, both associated with the NOD-like receptor signaling pathway, were upregulated in macrophages treated with *E. faecalis* MVs at pH 7.0 and 9.0. Significant differences were observed between the pH 9.0 *E. faecalis* MVs treatment and the neutral environment MVs (*P < 0.05, **P < 0.01, and ***P < 0.001). Furthermore, the *NOD2* gene was found to be upregulated following treatment with pH 9.0 *E. faecalis* MVs (*P < 0.05) (Fig. 10A through C). No statistically significant difference was noted in the expression levels of *NLRP3* and *PYCARD* (*P* > 0.05) (Fig. S3).

The NOD-like receptor family comprises pattern recognition receptors that are associated with stress-induced immune responses and inflammatory cell death, also known as pyroptosis (49). The findings indicated that pyroptosis-related genes *CASP1* and *IL1B* showed significant upregulation in both groups, with pH 9.0 *E. faecalis* MVs exhibiting a more marked effect. Additionally, *NFKB1* was significantly upregulated in the alkaline *E. faecalis* MV group (*P < 0.05 and ***P < 0.001) (Fig. 10D through F).

## DISCUSSION

AP constitutes an inflammatory response triggered by microbial infection within the root canal system (50). In the cases of PAP, *E. faecalis*, which exhibits a high detection rate, demonstrates pronounced resistance to chemical agents used in RCT, such as Ca(OH)_2_, NaClO, and CHX, in addition to mechanical preparation procedures (51–53). Consequently, this leads to the persistence of infection within the root canal. Extensive research has established that MVs are associated with bacterial survival and pathogenicity (25). A thorough investigation into the effects of MVs produced under various pH conditions on *E. faecalis*’s resistance to stressful environments, as well as the regulatory role in host immune responses and the underlying mechanisms, is essential for advancing our understanding of the pathogenic mechanisms of *E. faecalis* in PAP.

The findings from the environmental stress survival assay indicate that in highly alkaline environments with pH levels of 10.0 and 11.0, the addition of *E. faecalis* MVs produced under diverse pH conditions can augment the survival capacity of *E. faecalis*. Furthermore, the results demonstrate that the survival ability of *E. faecalis* in hyperosmolar environments can be enhanced by MVs. Our previous proteomic and metabolomic analyses revealed that the chaperone proteins DnaK and DnaJ are expressed in *E. faecalis* MVs under varying pH conditions (35). Proteins necessitate the assistance of chaperone proteins to preserve their proper conformation under stress conditions (54). The genes encoding DnaK and DnaJ are situated on the *dnaK* operon. Laport et al. (55) discovered that NaCl, SDS, and H_₂_O_₂_ can induce transcription of the *dnaK* operon in *E. faecalis*, indicating that DnaK and DnaJ are linked to *E. faecalis* resilience in stress environments. Moreover, both betaine and choline were identified in *E. faecalis* MVs at pH 7.0 and 9.0. These compounds serve as crucial protective agents for most microorganisms in response to hyperosmotic conditions (56, 57). Peddie et al. (58) demonstrated that the addition of betaine to hyperosmotic culture media results in a significant acceleration of *E. faecalis* growth rate.

Specifically, pH 9.0 *E. faecalis* MVs exhibited efficacy at higher concentrations of NaCl, whereas no statistical difference was observed in the group of *E. faecalis* MVs produced in a neutral environment. It is noteworthy that *E. faecalis* MVs function exclusively in more extreme oligotrophic environments, with a more pronounced effect observed at pH 9.0 *E. faecalis* MVs at high concentrations. The production of MVs in an alkaline environment may further enhance the survival of *E. faecalis* under conditions of high alkalinity, hyperosmotic stress, low nutrient environment, and CHX challenge. Our preliminary research indicated the upregulation of multiple stress proteins, including RelA_SpoT domain protein and Gls24 general stress protein, in the presence of pH 9.0 *E. faecalis* MVs (35). RelA and SpoT are ppGpp synthetases, with ppGpp being a global regulatory molecule that facilitates bacterial survival in stressful environments (59). As demonstrated by Giard et al. (60), the induction of the Gls24 protein in *E. faecalis* is triggered by glucose starvation. Furthermore, the survival of *gls24* gene mutant strains under starvation conditions is significantly reduced compared to that of wild-type strains. Consequently, we hypothesize that *E. faecalis* MVs may play a role in the survival of *E. faecalis* under stressful conditions, and that MVs produced in alkaline environments may be relevant in more extreme environments.

Root canal irrigation is a crucial procedure for eliminating microorganisms. CHX has been demonstrated to reduce *E. faecalis* in root canals and dentinal tubules effectively (61–63). According to the findings of Briseño-Marroquín et al. (64), the survival rate of *E. faecalis* was observed to be 0% after 5 min of CHX treatment. Furthermore, the study indicated that *E. faecalis* could not survive after 3 min of CHX exposure. However, following a 1-min treatment, lower concentrations of pH 7.0 *E. faecalis* MVs and higher concentrations of pH 9.0 *E. faecalis* MVs were found to significantly increase the survival rate of *E. faecalis*. The antibacterial mechanism of CHX is associated with its cationic properties, enabling it to form strong adsorption bonds with bacterial surfaces, thereby further compromising cellular integrity (61, 62). Our preliminary research indicated that pH 7.0 *E. faecalis* MVs possessed larger diameters (35). It is therefore hypothesized that the larger diameter of pH 7.0 *E. faecalis* MVs provides a greater surface area for the adsorption of CHX, thereby reducing CHX adhesion to bacteria and resulting in more effective protection. Furthermore, in our previous research, SalB was identified in *E. faecalis* MVs under different pH conditions (35). SalB has been shown to induce the activity of cephalosporin-related resistance factors in *E. faecalis* (65). We hypothesize that *E. faecalis* MVs are associated with the drug resistance of *E. faecalis*.

The present study indicates that in most stressful environments, the impact of *E. faecalis* MVs on the survival rate of *E. faecalis* does not increase proportionally with higher concentrations. A concurrent investigation has demonstrated that the addition of low concentrations of enterotoxigenic *E. coli* (ETEC) OMVs during polymyxin B treatment of ETEC results in a significant rise in bacterial survival rates and the development of resistance to polymyxin B (66). Conversely, elevated concentrations of OMVs have been shown to provide protective effects, although they do not confer resistance to infection. Therefore, it can be inferred that the protective effect of MVs may not be linearly related to their concentration. Nonetheless, further research is necessary to elucidate the exact mechanism by which *E. faecalis* MVs influence bacterial responses under stressful conditions.

Previous studies have documented divergent findings regarding the influence of *E. faecalis* infection on macrophage polarization (67). Wei et al. (68) demonstrated that carbohydrate metabolism in *E. faecalis* OG1RF can induce RAW264.7 macrophages to polarize toward the M1 phenotype. Conversely, Polak et al. (24) reported that *E. faecalis* ATCC V583 induces M2 polarization in THP-1 macrophages. Our preliminary research, along with the findings of other investigators, suggests that *E. faecalis* MVs significantly stimulate the secretion of pro-inflammatory cytokines, including IL-6, TNF-α, and IL-1β, by macrophages (33, 35). Furthermore, an elevated IL-1β/IL-1ra ratio was observed, suggesting a considerable pro-inflammatory effect exerted by *E. faecalis* MVs. Flow cytometry analyses further elucidated the impact of *E. faecalis* MVs produced under varying pH conditions on macrophage polarization. The findings revealed that MVs derived from *E. faecalis* in both neutral and alkaline environments markedly promote M1 polarization. These results align with prior observations by Ma et al. (31), who investigated the effects of MVs generated by *E. faecalis* under neutral conditions on RAW264.7 cells. Moreover, it was determined that MVs produced at pH 9.0 can also induce M1 polarization, indicating a potential link between these effects and the capacity of *E. faecalis* to sustain persistent inflammation in the alkaline milieu of the root canal following RCT. Our investigation further demonstrated that varying concentrations of *E. faecalis* MVs do not produce significant differences in their effects on macrophage polarization, implying that even low MVs concentrations can provoke inflammatory responses. Elizagaray et al. (69) identified that stimulation with 800 ng/mL of *Bordetella pertussis* OMVs induces caspase-11 and guanylate-binding protein-dependent non-canonical inflammasome activation. Moreover, the results indicated that MVs produced in a neutral environment exhibit a more robust pro-inflammatory response compared to those produced in an alkaline setting at equivalent concentrations. Building on previous research, which revealed that proteins differentially expressed in MVs produced under alkaline conditions are predominantly involved in metabolism, response to stimuli, and drug resistance, it is plausible to hypothesize that, under alkaline conditions, *E. faecalis* may enhance its adaptability to extreme environments via MVs.

ROS plays a pivotal role as a mediator in the immune responses of macrophages (70, 71). Polak et al. (24) observed that following the infection of macrophages with *E. faecalis*, the mitochondrial activity of surviving macrophages remained unchanged, while internal ROS levels increased. The present study demonstrated that *E. faecalis* MVs produced in neutral and alkaline environments can induce the generation of intracellular ROS in macrophages, and that this effect intensifies with increasing MVs concentration. Similarly, Xie et al. (72) provided evidence that *P. gingivalis* OMVs can infect gingival keratinocytes and induce ROS production in a dose-dependent manner, thereby promoting oxidative stress. An additional finding of the present study was that *E. faecalis* MVs at pH 9.0 could also stimulate macrophages to generate ROS, suggesting that *E. faecalis* can induce intracellular ROS production in macrophages via MVs to sustain inflammation in an alkaline environment post-treatment, which may be associated with PAP.

Subsequent analysis using RNA-seq was conducted to elucidate further the mechanisms by which *E. faecalis* MVs, produced under distinct pH conditions, influence macrophage immune responses. The findings indicated that *E. faecalis* MVs significantly modulate the transcriptional levels of numerous genes within macrophages. The DEGs and KEGG pathway enrichment analyses associated with the effects of pH 7.0 and pH 9.0 *E. faecalis* MVs on macrophages predominantly relate to inflammatory processes. Analysis of the KEGG pathway interaction network revealed that the cytokine-cytokine receptor pathway plays a critical role in these alterations. Most genes are connected to chemokines, including factors linked to M1 polarization (*CCL2*, *CCL3*, *CCL4*, and *CCL5*) (73–78), monocyte/macrophage chemokines (*CCL8* and *CXCL14*) (78), neutrophil chemokines (*CXCL1*, *CXCL2*, *CXCL6*, and *CXCL8*) (79), natural killer cell-associated cytokines (*CXCL10*) (80), and B lymphocyte chemokines (*CXCL13*) (81). Notably, the upregulation of *CXCL10* has been observed in human samples during the progression of apical periodontitis (82, 83). Furthermore, genes involved in inflammatory responses, such as *IL1B*, *IL7R*, *IL15RA*, *IL18R1*, *IL23A*, *IL32*, *TNF*, *TNFRSF9*, *TNFSF10*, and *TNFSF13B,* are also identifiable (84–91). Cruz et al. (91) demonstrated that BAFF, encoded by *TNFSF13B*, is a cytokine potentially involved in both acute and chronic apical periodontitis. These results suggest that both neutral and alkaline *E. faecalis* MVs stimulate macrophages to secrete chemokines and express receptors, thereby initiating a series of alterations in immune metabolism and inflammatory responses.

The results of the KDA indicate that numerous key genes, including *GBP1*, *MX2*, and *OAS1*, are implicated in the NOD-like receptor signaling pathway. RT-qPCR results corroborated the upregulation of pathway-associated genes such as *TNF* and *GBP1*. Similarly, Ma et al. (31) found that MVs produced by *E. faecalis* in a neutral environment promote macrophage polarization toward the M1 phenotype via the NOD2/RIPK2 signaling pathway. Notably, in the RT-qPCR analysis, NOD2 was significantly upregulated exclusively in the group exposed to alkaline *E. faecalis* MVs. The present study demonstrates that NOD-like receptor signaling plays a vital role in the inflammatory response of macrophages to *E. faecalis* MVs generated in an alkaline environment, with a more pronounced upregulation observed. Okugawa et al. (92) revealed that both NOD1 and NOD2 contribute to the recognition of periodontal pathogens through fragments of bacterial peptidoglycan. NOD2 has been shown to mediate NF-κB activation and the expression of the pro-inflammatory cytokine TNF-α, thereby initiating an immune response against pathogens. Prates et al. (93) reported that NOD2 is detectable in the periapical tissues of rats with mandibular first molar apical periodontitis, suggesting that the NOD2 receptor may contribute to the progression of bone resorption in experimental models of periodontitis. Consequently, the NOD-like receptor signaling pathway may be involved in the inflammatory response mechanisms mediated by *E. faecalis* MVs derived from an alkaline environment.

Pyroptosis is characterized as an inflammatory mode of cell death marked by the release of substantial inflammatory mediators following cell rupture. Wu et al. (94) identified elevated expression levels of caspase-1 in human apical periodontitis tissues relative to human periodontal ligament tissues. Our experimental data demonstrated that *E. faecalis* MVs produced under varying pH conditions could induce the upregulation of pyroptosis-associated genes in macrophages, with MVs at pH 9.0 exhibiting a more pronounced effect than those in a neutral environment. In the RNA-seq analysis, a significant upregulation of pyroptosis-related genes *IL1B* (3.17- and 3.94-fold) and *NFKB1* (1.11- and 1.41-fold) was observed following stimulation with neutral and alkaline *E. faecalis* MVs. These findings were corroborated by RT-qPCR results. Moreover, RT-qPCR revealed a significant upregulation of *CASP1*. Further investigation has also established that *F. nucleatum* OMVs possess the capacity to induce pyroptosis in macrophages (84). It is noteworthy that *E. faecalis* MVs produced in a neutral environment exert a more significant effect on ROS generation and M1 polarization phenotypes. Conversely, at the mRNA level, *E. faecalis* MVs at pH 9.0 demonstrated a more substantial pro-inflammatory effect, which warrants further exploration in terms of post-translational modifications.

It is noteworthy that among the DEGs induced by *E. faecalis* MVs from various environments in macrophages, a significant number were related to osteoclast differentiation, including *TNF*, *NFKB1*, *NFKBIA*, *RELB*, *NCF1*, *STAT1*, and *SOCS3* (95–101). Notably, the DEGs induced by pH 9.0 *E. faecalis* MVs demonstrated greater upregulation of osteoclast differentiation-associated genes compared to those in a neutral environment, such as *CSF1* and *SQSTM1*. Research has indicated that osteoclasts develop through the fusion of hematopoietic stem cell-derived monocyte precursors in the presence of CSF1 and RANK ligand, which are critical local signals for osteoclast survival and differentiation (102, 103). A study conducted by Virdee et al. (104) revealed elevated concentrations of CSF1 in over 75% of periapical tissue fluid samples obtained from patients diagnosed with apical periodontitis. *SQSTM1* is associated with osteoclast differentiation and mitochondrial autophagy (105). It is hypothesized that these factors may be involved in the bone resorption response induced by apical periodontitis. Further research is necessary to investigate the relationship between *E. faecalis* MVs and osteoclast differentiation.

Overall, the present study investigated the effects of *E. faecalis* MVs produced under varying pH conditions on the adaptation of *E. faecalis* to extreme environments and their influence on macrophage inflammatory responses. The results demonstrated that MVs from *E. faecalis* under different pH conditions facilitated the survival of *E. faecalis* in stressful environments and exhibited significant pro-inflammatory effects on macrophages, while also altering the transcriptional levels of macrophage genes. The present findings contribute to the existing body of knowledge regarding the relationship between *E. faecalis* and the host in the PAP. However, further research is required to investigate the *in vivo* effects of *E. faecalis* MVs and to explore their potential efficacy as therapeutic targets.

## MATERIALS AND METHODS

### Bacterial strain and growth conditions

*E. faecalis* strain OG1RF ATCC47077 was procured from the Guangdong Detection Center of Microbiology (GDDCM) and propagated in tryptic soy broth (Huankai Biology, Guangzhou, China) under anaerobic conditions at 37°C. The pH of the TSB was calibrated to 7.0 (neutral) and 9.0 (alkaline) using KH_2_PO_4_ and NaOH and then sterilized via filtration using a 0.22 µm pore size filter unit (Millipore, Millex-GP, Burlington, MA, USA).

### Preparation and identiﬁcation of *E*. *faecalis* MVs

*E. faecalis* MVs were prepared through centrifugation and purified via density gradient centrifugation, as previously described (35). *E. faecalis* was cultured for 10 h in 1 L of TSB at pH 7.0 and 9.0, respectively. Enrichment of *E. faecalis* MVs from the culture supernatant was accomplished using sequential centrifugation. The culture broth was centrifuged at 10,000 × *g* for 30 min at 4°C to remove bacterial precipitates. Subsequently, the supernatant was sterilized employing a 0.22 µm pore size sterile filter (Millipore, Millex-GP, Burlington, MA, USA). The sterile supernatant was concentrated utilizing a 100 kDa ultrafiltration tube (Millipore, Millex-GP, Burlington, MA, USA), followed by centrifugation at 4,000 × *g* for 30 min at 4°C. The concentration in the upper chamber was then subjected to ultracentrifugation using the Optima XE-100 ultracentrifuge (Beckman, Brea, CA, USA). MV precipitates were obtained by ultracentrifugation at 150,000 × *g* for 2 h at 4°C. The MVs were washed with PBS and subsequently collected through ultracentrifugation. The precipitated MVs were resuspended in 40% OptiPrep (Sigma-Aldrich, Darmstadt, Germany), sequentially layered with 35%, 30%, 25%, and 20% OptiPrep solutions. After centrifugation at 200,000 × *g* for 120 min at 4°C, the MVs situated between the 25% and 30% OptiPrep layers were collected. These MVs were resuspended in PBS and further centrifuged at 100,000 × *g* for 70 min at 4°C. The precipitated MVs were then collected and resuspended in 1 mL of PBS. To confirm the sterility of the MVs, they were inoculated onto TSB agar plates and incubated for 24 h. The protein concentration of *E. faecalis* MVs was measured using the BCA protein quantification kit (Beyotime, Shanghai, China). GDDCM was commissioned to conduct TEM observations of *E. faecalis* MVs. A volume of 5 µL of MV samples from neutral and alkaline environments was dispersed and suspended using an ultrasonic dispenser. These MVs were subsequently deposited onto a polymethyl vinyl acetate lipid support membrane positioned on a 75-mesh copper grid. The samples were incubated for 5 min and were then washed four times with pure water. A mixture of 2% methylcellulose and 3% uranyl acetate was prepared in a 9:1 ratio. The samples underwent negative staining by being placed in an ice bath for 2 min, followed by air drying at room temperature. The MVs were observed and imaged using an H-7650 transmission electron microscope (Hitachi, Tokyo, Japan).

### Environmental stress survival assay

The survival capacity of *E. faecalis* before and after the addition of *E. faecalis* MVs under environmental stress was compared by calculating colony-forming units (CFUs) using the plate count method. The components of the bacterial culture medium were then conditionally adjusted to establish various stress models. The environmental stresses employed in this study included alkalinity, hyperosmosis, starvation, and exposure to CHX. Specifically, the alkaline environment was generated by adjusting the pH of TSB to 10.0 and 11.0 using 1 M KH_₂_PO_₄_. Before adjustment, the pH of the TSB was verified to be neutral and served as CFU_T0_. The hyperosmotic environment was created by adjusting the NaCl concentration in TSB to 4% and 8%. TSB containing 0% NaCl was designated as CFU_T0_. For nutrient-deficient conditions, TSB was diluted at ratios of 1:2 (50% TSB) and 1:10 (10% TSB), with undiluted TSB (100%) serving as CFU_T0_. *E. faecalis* was collected during the logarithmic growth phase, and *E. faecalis* MVs produced under different pH conditions at concentrations of 0, 0.5, 1.0, and 5.0 µg/mL, respectively, were added to the respective culture media. The bacteria were then cultured at 37°C for 2 h. The CFU at this point was calculated and designated as CFU_T1_. Subsequently, to evaluate the resistance of *E. faecalis* to CHX, MVs were added to the bacterial cultures and incubated for 2 h, followed by exposure to 0.2% and 2% CHX (Solarbio, Beijing, China) for 1 and 3 min, respectively, as CFU_T1_. TSB containing 0% CHX was designated as CFU_T0_. After washing with PBS, serial dilutions were performed, and CFUs were enumerated using agar plate cultivation. The identification of *E. faecalis* colonies was facilitated by observing their bacterial morphology; colonies appeared white, round, and smooth. Identification was further confirmed by light microscopy following Gram staining. The ratio CFU_T1_/CFU_T0_ was recorded as the survival rate of *E. faecalis*, allowing for the analysis of whether MVs influence the resistance of *E. faecalis* to stress environments. The experiment was conducted in triplicate.

### Culture and differentiation of THP-1 cells

The human acute monocytic leukemia cell line (THP-1) was supplied by the Stem Cell Bank of the Chinese Academy of Sciences (Shanghai, China). THP-1 cells were cultured routinely in RPMI 1640 medium (Thermo Fisher Scientific Inc., Waltham, MA, USA), supplemented with 10% fetal bovine serum (FBS) (Australian origin, Thermo Fisher Scientific Inc., Waltham, MA, USA), and maintained at 37°C within a humidified incubator with 5% CO_₂_. For macrophage differentiation, THP-1 cells were exposed to a concentration of 50 ng/mL of phorbol 12-myristate 13-acetate (Macklin Inc., Shanghai, China) for 48 h. Subsequently, the dTHP-1 cells were cultured in fresh RPMI 1640 medium supplemented with 10% FBS for an additional 24 h under a humidified atmosphere of 5% CO_₂_.

### Fluorescence flow cytometric analysis of cell surface markers

The dTHP-1 cells were seeded at a density of 1.0 × 10^6^ cells per well in a flat-bottom 6-well cell culture plate and incubated for 12 h before further application. The exposure of dTHP-1 cells to *E. faecalis* MVs derived from cultures with varying pH levels at concentrations of 0.5 and 1.0 µg/mL, respectively, was conducted for 24 h. PBS served as a negative control, while LPS at a concentration of 0.5 µg/mL was employed as a positive control. After a 24-h infection period with *E. faecalis* MVs produced under different pH conditions, the dTHP-1 cells were harvested for the phenotypic identification of macrophages. The cells were then washed with ice-cold fluorescence-activated cell sorting (FACS) buffer (1% BSA-PBS) and stained with APC-labeled anti-CD80 (BioLegend, Inc., San Diego, CA, USA) and PE-labeled anti-CD206 (BioLegend, Inc., San Diego, CA, USA) for 40 min at 4°C. Subsequent washing with FACS buffer was performed to remove unbound antibodies, and the samples were analyzed using a CytoFLEX flow cytometer (Beckman Coulter, Brea, CA, USA). Data analysis was conducted with FlowJo software (version 10.0). The assay was conducted across three independent experiments.

### ROS determination

Following a 24-h incubation period with *E. faecalis* MVs produced under varying pH conditions, the cell culture medium was aspirated. Serum-free medium was introduced and carefully pipetted to detach the adherent cells. As a positive control, cells treated with 48 µM of H_₂_O_₂_ were employed, while uninfected cells served as a negative control. The cells underwent two washes with serum-free medium, followed by staining with 10 µM of the oxidation-sensitive probe 2′7′-dichlorofluorescein diacetate (Applygen, Beijing, China) at 37°C in the dark for 40 min. The cells were then washed twice with PBS and resuspended in PBS to quantify the intracellular ROS production using a CytoFLEX flow cytometer paired with FlowJo software. Additionally, fluorescence intensity was measured at an excitation wavelength of 502 nm and an emission wavelength of 530 nm utilizing a fluorescence plate reader (BioTek Instruments, Inc., Winooski, VT, USA). Fluorescence observation was performed through a fluorescence microscope (Zeiss, Oberkochen, Germany). The experiment was conducted in triplicate.

### RNA extraction

Total RNA from dTHP-1 cells exposed to *E. faecalis* MVs derived from cultures under different pH conditions was extracted using RNAzolRT (Molecular Research Center Inc., Cincinnati, OH, USA). The RNA was then dissolved in 30 µL of RNase-free water. RNA quantification was performed with a NanoDrop One Microvolume Spectrophotometer (Thermo Fisher Scientific Inc., Waltham, MA, USA).

### RNA sequencing

The RNA-seq was conducted utilizing the DNBSEQ platform. SOAPnuke (version 1.6.5) was used for filtering reads and acquiring high-quality reads. Reads containing adapters, with a proportion of unknown bases exceeding 1%, and low-quality reads were eliminated. The resulting clean reads were subsequently aligned to the human reference genome (GRCh38.p12) using HISAT2 (version 2.0.4). Gene expression levels were quantified using the Bowtie2 (version 2.4.5) and RSEM (version 1.3.1) software packages. Stringent criteria, including |Log2 fold change| ≥ 1 and false discovery rate < 0.001, were applied to identify DEGs. The subsequent data analysis and figure generation involved GO (http://www.geneontology.org/) and KEGG (https://www.kegg.jp/) enrichment analyses, PPI analysis, and KDA of DEGs. These analyses were performed using the BGI Genomics data mining system, Dr.Tom (http://report.bgi.com). Additionally, the STRING database (version 12) was utilized to analyze gene network interactions among the selected DEGs. RT-qPCR was conducted to validate the RNA-seq findings. The primer sequences used are detailed in Table S4. The assay was conducted across three independent experiments.

### RT-qPCR

The application of RT-qPCR is employed to validate changes in mRNA expression levels. Total RNA was extracted and analyzed from dTHP-1 cells treated with *E. faecalis* MVs produced under varying pH conditions, utilizing the methodology previously described. Reverse transcription of RNA was conducted using PrimeScript RT Master Mix (Perfect Real Time) (TAKARA, Shiga, Japan), following the manufacturer’s instructions. Subsequently, RT-qPCR was performed using SYBR Premix Ex Taq II (Tli RNaseH Plus) (TAKARA, Shiga, Japan) on the LightCycler 96 system (Roche, Basel, Switzerland) to quantify relative mRNA expression levels. The 2^−ΔΔCt^ method was employed for data analysis, with normalization against the reference gene *GAPDH*. The primer sequences, specific to each gene, are provided in Table S4. The experiment was done in triplicate.

### Statistical analysis

Statistical analyses and illustrations were executed utilizing GraphPad Prism (version 9.5). In addition to the transcriptomics analysis, statistical significance (*P* < 0.05) was ascertained through one-way analysis of variance with Bonferroni’s multiple comparison correction or Student’s *t*-test. All experiments were performed on a minimum of three independent occasions.

## Data Availability

The RNA-seq raw data of this study have been submitted to the Sequence Read Archive (SRA) of the National Center for Biotechnology Information (NCBI) under accession number PRJNA1302447.

## SUPPLEMENTAL MATERIAL

Supplemental figures and tables jb.00377-25-s0001.pdf Figures S1 to S6 and Tables S1 to S4.

ASM does not own the copyrights to Supplemental Material that may be linked to, or accessed through, an article. The authors have granted ASM a non-exclusive, world-wide license to publish the Supplemental Material files. Please contact the corresponding author directly for reuse.
