# Supplementary material for: Effect of membrane vesicles produced under different pH conditions on the ability of Enterococcus faecalis to tolerate stressful environments and macrophages’ inflammatory response
Source: J Bacteriol. 2025 Oct 16;207(11):e00377-25. doi: 10.1128/jb.00377-25 (PMC12632260; doi:10.1128/jb.00377-25)
Supplement: Supplemental figures and tables — Figures S1 to S6 and Tables S1 to S4. [file jb.00377-25-s0001.pdf]

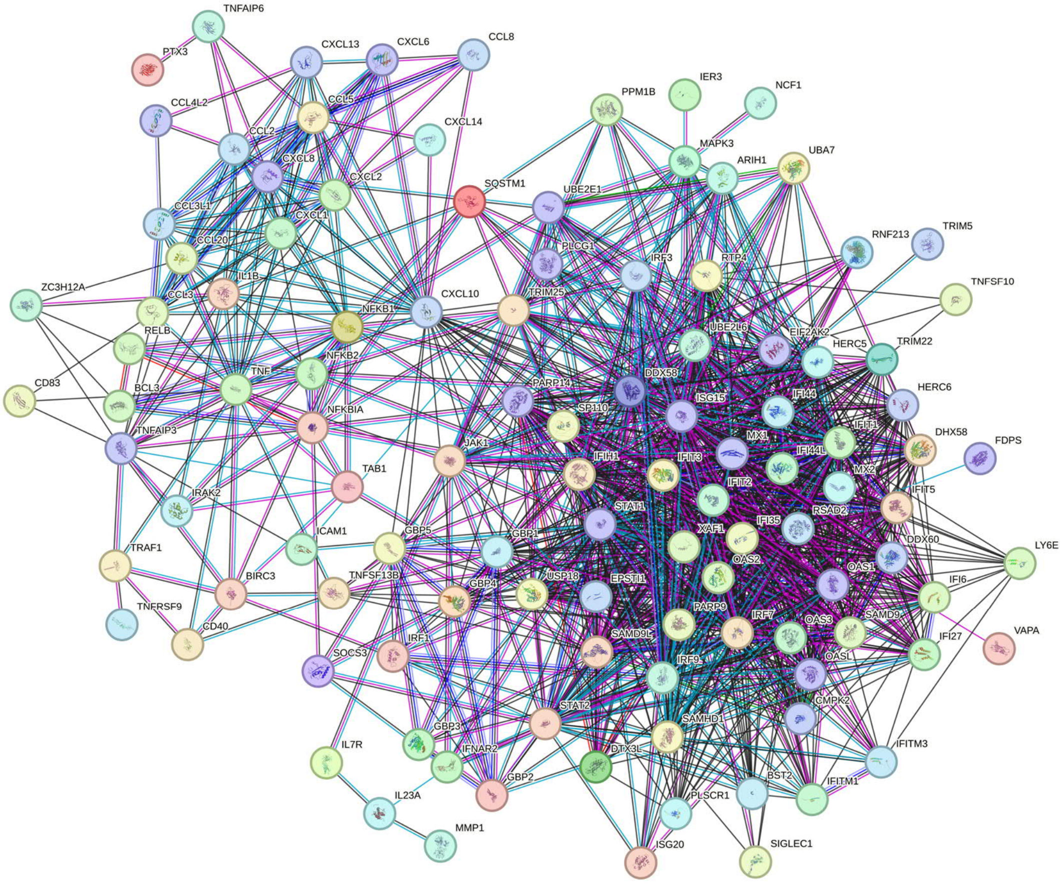

Figure S1. Analysis of the protein–protein Interaction (PPI) network of differentially expressed genes (DEGs) in macrophages infected with pH 7.0 *E. faecalis* MVs compared to the control.

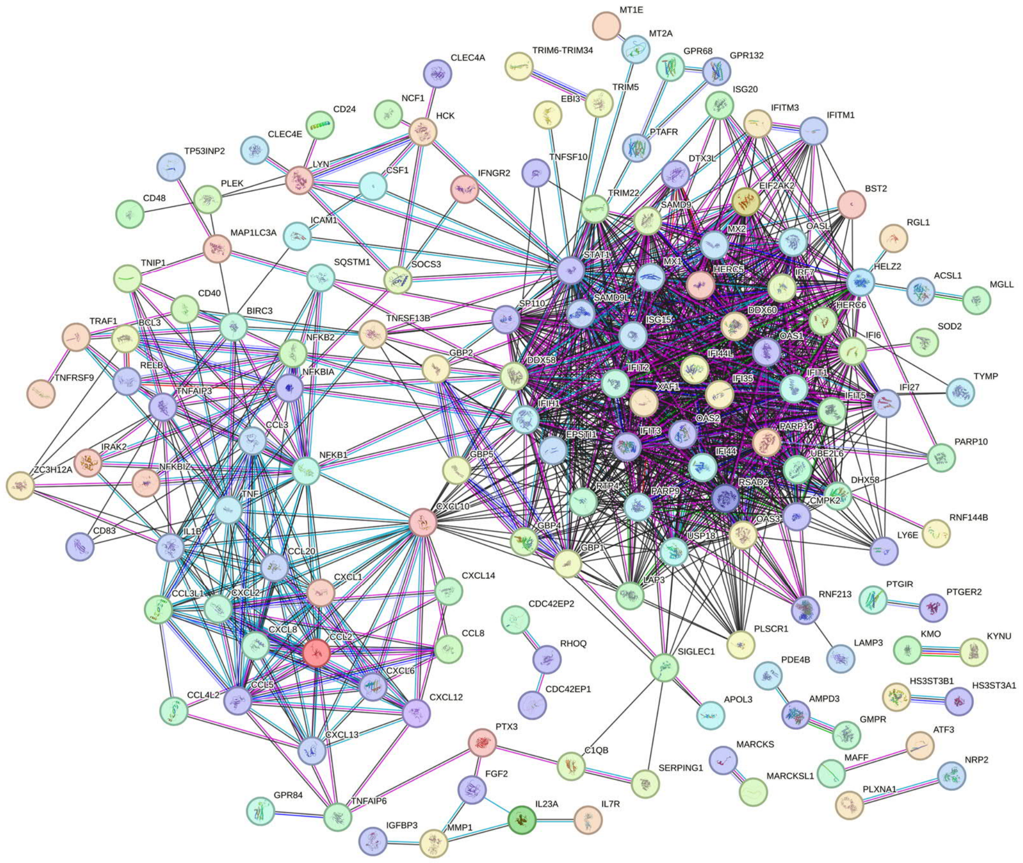

Figure S2. Protein–protein interaction (PPI) network analysis of differentially expressed genes (DEGs) in macrophages infected with pH 9.0 *E. faecalis* MVs compared to the control.

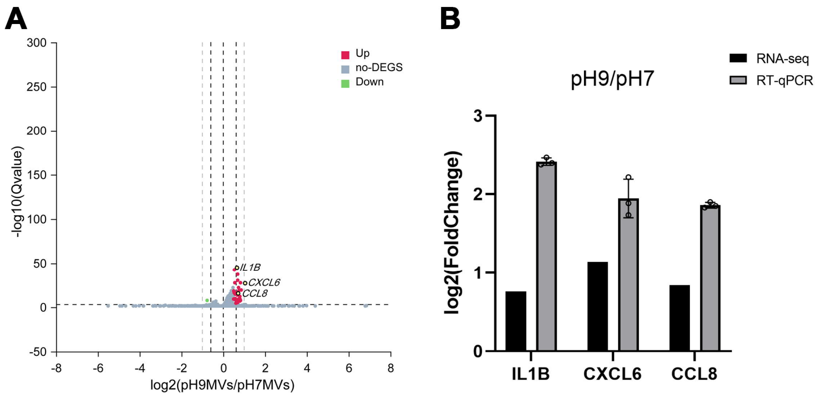

Figure S3. Differentially expressed genes (DEGs) in macrophages treated with pH 9.0 *E. faecalis* MVs in comparison to those treated with pH 7.0 *E. faecalis* MVs. (**A**) Volcano plots depict the DEGs, with each point representing a gene. Genes with a Log2 fold change ≥ 0.585 are highlighted in red to denote upregulation, while those with a Log2 fold change ≤ -0.585 are highlighted in green to denote downregulation (false discovery rate < 0.001). Grey indicates no significant difference. (**B**) Validation of the DEGs identified by RNA sequencing through RT-qPCR. The experiment was conducted in triplicate. The data are presented as mean with SD.

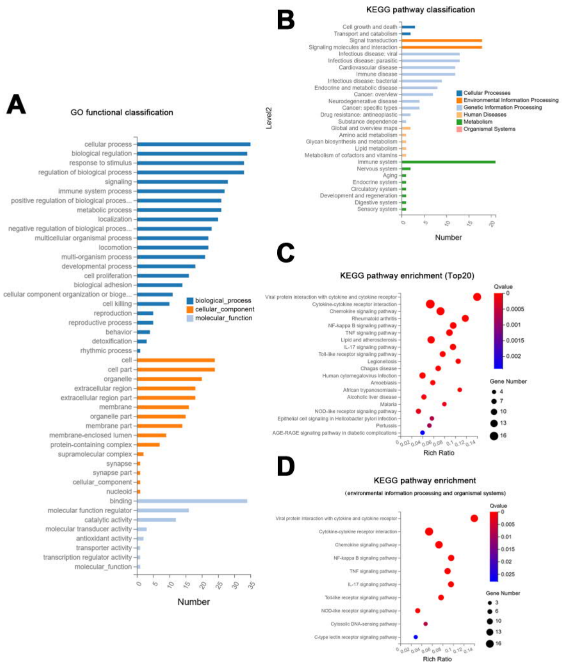

Figure S4. Analysis of Differentially Expressed Genes (DEGs) in macrophages exposed to pH 9.0 *E. faecalis* MVs in comparison to the pH 7.0 *E. faecalis* MVs group (|Log2 fold change| ≥ 0.585). (**A**) GO functional classifications. (**B**) Significantly enriched KEGG pathway classifications. (**C**) Bubble chart presenting the top 20 KEGG pathways enriched in the analysis. (**D**) Bubble chart displays KEGG pathway enrichment related to environmental information processing and organismal systems.

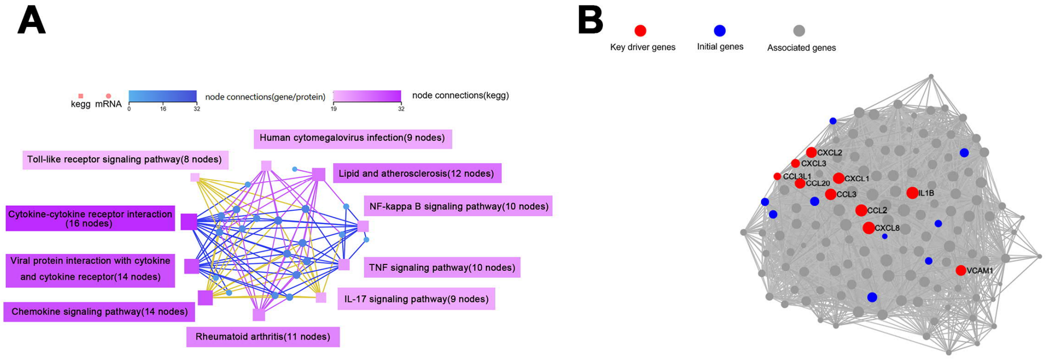

Figure S5. Network interaction analysis of KEGG pathways (**A**) and key driver analysis for protein-protein interactions (**B**), both of which were performed on the DEGs identified in macrophages treated with pH 9.0 *E. faecalis* MVs in comparison to the pH 7.0 *E. faecalis* MVs group (|Log2 fold change| ≥ 0.585).

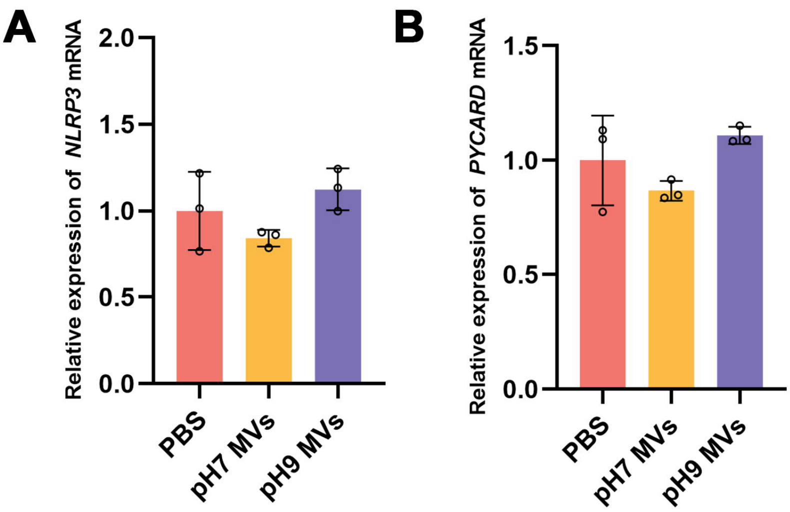

Figure S6. Validation of RT-qPCR results concerning the influence of *E. faecalis* MVs produced under varying pH conditions on *NLRP3* (**A**) and *PYCARD* (**B**). The assay was conducted in triplicate. Statistical analysis was performed using one-way analysis of variance for multiple comparisons, with Bonferroni's post hoc correction. Error bars denote the mean with SD. *$p < 0.05$, **$p < 0.01$, ***$p < 0.001$.

Table S1. DEGs in macrophages infected with pH 7.0 *E. faecalis* MVs vs Control (|Log2 fold change| ≥1)

Gene ID	Gene Symbol	log2 (pH7MVs / Control)	Qvalue (pH7MVs / Control)	pH7MVs Average FPKM	Control Average FPKM
100	'ADA'	1.635478683	2.89982330822354E-51	70.55	23.82666667
339416	'ANKRD45'	1.371951665	7.15463901809025E-14	5.736666667	2.323333333
80833	'APOL3'	1.128332759	1.28740452747229E-6	4.183333333	2.06
558	'AXL'	1.0969952	3.68715817103278E-37	57.86	28.42
116071	'BATF2'	1.700827141	2.7788547472360697E-13	4.543333333	1.49
597	'BCL2A1'	1.341264539	1.2620999012861401E-20	43.00666667	17.73
602	'BCL3'	1.884860245	2.2188163743926203E-52	37.73666667	10.75333333
330	'BIRC3'	2.313125912	2.26289878127175E-76	13.02666667	2.786666667
154743	'BMT2'	1.087236869	8.649375216482141E-19	10.42666667	5.143333333
684	'BST2'	1.071343604	3.2356088279371097E-16	735.8033333	365.5766667
713	'C1QB'	1.0501674	8.3149904659504E-10	16.92666667	8.563333333
838	'CASP5'	1.651955811	1.30227194411296E-11	7.4	2.46
6347	'CCL2'	3.686805484	2.73786487088795E-296	389.0066667	31.62333333
6364	'CCL20'	2.84354717	1.4422658931730802E-63	48.4	7.05
6348	'CCL3'	2.852239274	5.3441495493287306E-105	220.8633333	31.97
6349	'CCL3L1'	3.973796008	9.03857801704397E-59	34.79666667	2.37
414062	'CCL3L3'	3.710277491	1.5788203556381098E-138	113.4466667	9.123333333
6351	'CCL4'	4.560559637	1.9835569549636799E-268	349.0233333	15.50333333
9560	'CCL4L2'	5.13045818	3.16003885007928E-243	278.38	8.393333333
6352	'CCL5'	1.513138353	3.5157047731845804E-47	224.76	82.58666667
6355	'CCL8'	4.435602129	4.20474856078966E-126	66.19666667	3.203333333
100133941	'CD24'	1.578011357	1.8701547889759198E-26	11.66666667	4.02
958	'CD40'	1.474439646	2.9274395199389198E-43	54.93666667	20.73
962	'CD48'	1.827874386	4.9055896654003194E-36	45.25333333	13.31333333
9308	'CD83'	1.227681026	2.0816278717583298E-29	28.88	12.91333333
11135	'CDC42EP1'	1.115744695	4.11761533168347E-8	6.08	2.943333333
10435	'CDC42EP2'	1.078072832	6.30621415410255E-5	3.416666667	1.696666667
9435	'CHST2'	1.006356258	4.42767423113532E-30	29.08	15.18
50856	'CLEC4A'	1.148709783	3.3586305516102E-11	14.13333333	6.7
26253	'CLEC4E'	2.704253069	3.2754391462009097E-62	13.57333333	2.18
129607	'CMPK2'	2.295362736	1.4515892522641E-77	29.20666667	6.246666667
51232	'CRIM1'	1.547392056	9.6782885546427E-35	19.51333333	7.046666667
2919	'CXCL1'	2.457784438	4.1164433489356E-115	77.98666667	14.87333333
3627	'CXCL10'	3.344725783	1.45756449713142E-172	98.88333333	10.18666667
10563	'CXCL13'	4.050248944	4.71458264298042E-68	21.27	1.346666667
9547	'CXCL14'	2.147300063	2.75159960444114E-41	18.89666667	4.473333333
2920	'CXCL2'	2.703789478	6.51613963962457E-54	29.12	4.676666667
6372	'CXCL6'	2.112283275	9.12629315917465E-37	14.60666667	3.543333333
3576	'CXCL8'	3.542493654	0	552.2466667	49.42333333
140947	'DCANP1'	1.773876197	7.79087475397555E-39	37.39333333	11.47
55601	'DDX60'	1.510044303	9.41921485190959E-45	17.39	6.403333333
79132	'DHX58'	1.122949091	2.83266297536339E-17	15.78666667	7.593333333
54567	'DLL4'	1.812917954	8.73793183486582E-49	12.48666667	3.733333333
126820	'DNAI3'	1.171641833	1.93281142911263E-9	4.743333333	2.203333333
55332	'DRAM1'	1.278539578	1.2536545604908201E-46	34.04666667	14.82333333
151636	'DTX3L'	1.061305084	4.98433206599112E-29	32.66666667	16.44333333
1844	'DUSP2'	1.104059081	1.00301289102233E-6	5.576666667	2.713333333
10148	'EBI3'	2.203876815	4.55395473236937E-113	204.93	46.56
10938	'EHD1'	1.555371205	1.14260055159921E-60	26.68	9.513333333
5610	'EIF2AK2'	1.256104242	1.37011684379746E-30	31.72666667	13.96
5168	'ENPP2'	1.302313989	3.10229257652658E-7	2.356666667	1.006666667
94240	'EPSTI1'	1.805840087	5.03170627660553E-69	30.19333333	8.913333333
91010	'FMNL3'	1.265819347	1.56604558674379E-26	6.263333333	2.66
53826	'FXYD6'	1.058425147	1.41318241064649E-14	24.09333333	12.07333333
2633	'GBP1'	2.12148388	2.1918871370037098E-82	22.42666667	5.403333333
115361	'GBP4'	1.64343454	7.27746129160863E-34	5.13	1.726666667
115362	'GBP5'	2.329775117	5.53826906102847E-82	6.923333333	1.446666667
2643	'GCH1'	1.524854659	9.67305248007648E-13	3.423333333	1.366666667
169792	'GLIS3'	1.557885001	3.96103760620015E-27	3.226666667	1.106666667
2766	'GMPR'	1.550635828	2.06022410881498E-6	3.583333333	1.28
53831	'GPR84'	1.316955286	4.85317061306726E-19	15.43666667	6.456666667
85441	'HELZ2'	1.301379644	5.0746851743770396E-40	13.78333333	5.873333333
51191	'HERC5'	1.030925009	1.94565479378697E-24	22.81666667	11.71333333
55008	'HERC6'	1.857157002	5.9540629496817205E-68	27.64666667	7.973333333
3081	'HGD'	1.192443435	1.57255429597353E-6	5.546666667	2.576666667
3096	'HIVEP1'	1.123029903	3.54101248240136E-13	3.226666667	1.56
9955	'HS3ST3A1'	1.061881719	1.74979563386789E-7	2.44	1.24
9953	'HS3ST3B1'	1.665339772	1.67660928484122E-20	3.116666667	1.03
3383	'ICAM1'	1.561873579	3.49022492886765E-97	210.4566667	74.75333333
23308	'ICOSLG'	1.541097747	9.04909012749922E-59	11.78666667	4.33
8870	'IER3'	2.243954424	5.7240760446585E-48	27.09333333	5.98

3429	'IFI27'	2.246718793	2.23956060848336E-51	1062.623333	235.5633333
3430	'IFI35'	1.340470805	2.51829630472934E-32	165.0733333	68.15
10561	'IFI44'	2.15011766	1.83734651572509E-90	137.3166667	32.21333333
10964	'IFI44L'	3.669545209	8.936313377713569E-265	40.47	3.336666667
2537	'IFI6'	2.275949601	1.29706377558942E-84	1581.966667	341.06
64135	'IFIH1'	1.226419173	1.36302089757185E-48	65.43333333	29.21666667
3434	'IFIT1'	2.213920813	3.56785314462422E-68	20.09	4.526666667
3433	'IFIT2'	2.026377012	1.39616885587432E-50	10.81666667	2.786666667
3437	'IFIT3'	2.192473447	8.290771079801269E-94	36.31333333	8.326666667
24138	'IFIT5'	1.354976298	4.4810374986732705E-15	4.033333333	1.653333333
8519	'IFITM1'	3.263219281	7.230272695268451E-154	1097.633333	119.4233333
10410	'IFITM3'	2.096970997	1.50200473629873E-75	2254.763333	550.4566667
3486	'IGFBP3'	1.979411337	4.55587164484848E-139	525.3633333	139.5933333
3601	'IL15RA'	1.478517417	7.58952930955329E-8	4.016666667	1.476666667
8809	'IL18R1'	2.380063996	1.06028846235413E-46	6.013333333	1.243333333
3553	'IL1B'	3.172305708	3.9297221148310006E-213	117.7	13.68666667
51561	'IL23A'	1.120958129	1.01833139800427E-7	14.22333333	6.83
9235	'IL32'	2.607929803	1.20588267580568E-71	111.31	19.21
259307	'IL4I1'	1.691167986	3.92368099038725E-73	302.2333333	98
3575	'IL7R'	1.619874143	8.1771014729911805E-25	5.776666667	1.97
3656	'IRAK2'	2.502488765	2.24370158013877E-62	9.253333333	1.71
3665	'IRF7'	1.557906054	1.00197771495224E-36	39.72	14.33
9636	'ISG15'	2.958262046	6.57218243595542E-22	528.0566667	70.79666667
3669	'ISG20'	1.171692494	3.46346153894611E-6	5.383333333	2.48
3672	'ITGA1'	1.461527088	1.54305655722072E-24	2.693333333	1.023333333
8564	'KMO'	1.093745219	5.65627209305976E-22	8.673333333	4.273333333
8942	'KYNU'	1.380802749	5.88546371306E-68	156.4133333	59.45333333
27074	'LAMP3'	3.112768408	1.63424146150617E-80	9.49	1.156666667
51056	'LAP3'	1.045763077	1.13100790990462E-41	261.5466667	132.6933333
3959	'LGALS3BP'	1.290329353	1.85478778479456E-35	32.15333333	13.78
102723996	'LOC102723996'	1.545473722	3.8818220002384094E-42	17.87666667	5.983333333
4061	'LY6E'	2.413117045	1.6146981422143599E-134	670.7766667	131.7733333
4067	'LYN'	1.129561996	1.03584112094317E-48	139.7666667	67.02333333
23764	'MAFF'	1.211619982	6.99997482937337E-27	19.33666667	8.706666667
84557	'MAP1LC3A'	1.547151373	1.0171108326526901E-17	25.61333333	9.183333333
4082	'MARCKS'	1.095257286	4.38082340265913E-33	104.8866667	51.56
255231	'MCOLN2'	1.143495147	4.8481355890244E-38	52.90333333	25.11
4223	'MEOX2'	1.361987272	8.97667641916908E-8	2.95	1.2
11343	'MGLL'	1.365560121	1.65085852992601E-35	18.66666667	7.633333333
4312	'MMP1'	1.73383042	4.19811324581266E-21	7.92	2.523333333
4321	'MMP12'	2.132755285	3.66718284960073E-22	6.823333333	1.63
64386	'MMP25'	1.237132373	1.2743248184891501E-19	9.266666667	4.1
9242	'MSC'	1.76851213	1.9871339629918597E-43	30.81666667	9.466666667
4502	'MT2A'	1.669255408	5.56003945081483E-19	147.35	48.59666667
4509	'MT-ATP8'	1.304946467	1.345082140826E-4	4544.71	2231.42
4599	'MX1'	2.32820013	9.63086100585873E-168	96.22	20.03333333
4600	'MX2'	1.963133697	5.94656828869658E-136	73.41	19.77
653361	'NCF1'	1.556574053	1.2445331411564898E-55	267.9533333	95.33666667
4790	'NFKB1'	1.117257559	2.3040386122697397E-33	43.75	21.19
4791	'NFKB2'	1.252543729	3.8542153223718795E-43	35.46666667	15.68666667
4792	'NFKBIA'	1.319855016	1.33174988716777E-50	331.24	138.92
140688	'NOL4L'	1.130182477	2.2353585200923803E-19	7.133333333	3.443333333
10062	'NR1H3'	1.583866093	5.38611377703369E-53	73.19666667	26.75666667
4938	'OAS1'	1.405935642	1.41271858057353E-50	155.7666667	61.38666667
4939	'OAS2'	1.917822921	7.43916169905837E-85	111.7	31.92
4940	'OAS3'	2.135088206	5.6870203932092E-100	102.7466667	24.55333333
8638	'OASL'	2.051851749	8.07268982989647E-52	12.24333333	3.1
440836	'ODF3B'	1.286460031	1.3014184564768699E-12	20.51	8.613333333
5027	'P2RX7'	1.117131703	5.496810183205711E-20	13.14666667	6.323333333
84875	'PARP10'	1.083801226	1.23166065846571E-29	56.04	27.66333333
54625	'PARP14'	1.358894327	2.14619054373867E-53	30.52666667	12.49333333
83666	'PARP9'	1.556037537	9.213171467581911E-76	63.17666667	22.54666667
5142	'PDE4B'	1.206764446	1.1825808862527301E-20	9.21	4.203333333
9659	'PDE4DIP'	1.076842666	8.50123839950912E-28	12.7	5.906666667
11040	'PIM2'	1.292774858	1.04651413542281E-42	43.47	18.59666667
11142	'PKIG'	1.668911035	4.53002182523118E-33	30.68	10.27
5359	'PLSCR1'	1.242001818	3.3182121210110904E-44	158.8366667	70.16333333
9050	'PSTPIP2'	1.473016609	2.6064596394340697E-40	18.62666667	7.036666667
5724	'PTAFR'	1.488785117	8.42009191581504E-67	31.42666667	11.74666667
5732	'PTGER2'	1.184065693	1.95259406644294E-7	3.603333333	1.663333333
5739	'PTGIR'	1.615431481	2.695718290105E-18	6.8	2.383333333
5806	'PTX3'	1.335061686	1.3504401587592401E-31	24.97333333	10.38
25797	'QPCT'	1.351682387	1.89598514522408E-23	17.46	7.176666667
153020	'RASGEF1B'	1.336949304	1.8950952133326E-11	4.043333333	1.673333333

5971	'RELB'	1.144217499	4.28007010767596E-31	43.55	20.64333333
23180	'RFTN1'	1.420625303	1.49398204861218E-44	26.71	10.53666667
23179	'RGL1'	1.234473522	2.20456263186375E-43	43.62333333	19.46
6004	'RGS16'	1.157846855	2.37743635662755E-13	9.803333333	4.603333333
23586	'RIGI'	1.580439786	1.13309304365511E-57	16.60333333	5.81
255488	'RNF144B'	1.963766054	1.4845315654927501E-52	10.78333333	2.916666667
57674	'RNF213'	1.041041643	3.52299444759669E-28	17.25333333	8.706666667
91543	'RSAD2'	2.116121096	3.85438007163572E-69	14.25	3.463333333
54809	'SAMD9'	1.252418784	3.65943935847828E-27	19.17333333	8.46
219285	'SAMD9L'	1.534359183	6.20074543484579E-37	22.80333333	8.28
64218	'SEMA4A'	1.106271666	1.7521475762309E-41	86.24666667	41.98666667
710	'SERPING1'	1.417268534	1.87519866539906E-11	6.273333333	2.46
285590	'SH3PXD2B'	1.130750083	1.12008932264068E-24	7.266666667	3.506666667
6614	'SIGLEC1'	1.769335709	5.07856861879544E-80	36.15333333	11.13666667
57823	'SLAMF7'	2.564866283	2.38341001016213E-176	72.32333333	13.06666667
9120	'SLC16A6'	1.24083007	7.05311283338102E-8	2.416666667	1.073333333
11182	'SLC2A6'	1.260326616	1.40570907327544E-53	87.12	38.48
9021	'SOCS3'	1.541148742	2.82662349668503E-27	11.27666667	4.083333333
6648	'SOD2'	2.402226313	6.59275571678021E-180	527.5733333	112.6933333
3431	'SP110'	1.038859705	7.48265459132787E-32	46.83666667	24.41
26228	'STAP1'	1.618176866	8.430522052217989E-11	4.593333333	1.503333333
6772	'STAT1'	1.788337687	1.2496444331066602E-98	202.5466667	61.06
6819	'SULT1C2'	1.593797879	6.00538285700191E-10	2.99	1.04
497189	'TIFAB'	2.001282926	2.3727090811602698E-86	29.37	7.7
134285	'TMEM171'	1.520337859	1.777212784904E-4	2.92	1.066666667
7124	'TNF'	2.221268708	9.583313027263759E-86	62.85666667	14.13
7127	'TNFAIP2'	1.448078406	1.4157489012644099E-56	66.74	25.59333333
7128	'TNFAIP3'	1.21493232	4.25887643142303E-41	124.5066667	56.32333333
7130	'TNFAIP6'	3.981934055	2.23788129344554E-234	82.01333333	5.44
3604	'TNFRSF9'	2.453964638	9.14761339976028E-99	22.57666667	4.636666667
8743	'TNFSF10'	1.618382115	4.97633546709302E-44	26.68666667	9.136666667
10673	'TNFSF13B'	1.573304349	1.31342770831257E-29	14.35666667	5.013333333
58476	'TP53INP2'	1.599498654	7.49195917185176E-37	9.516666667	3.29
7185	'TRAF1'	2.725352086	8.121242446541161E-91	13.18666667	2.036666667
10346	'TRIM22'	2.115276355	5.70132751479034E-75	59.12666667	14.33666667
53840	'TRIM34'	1.078494037	2.1225792987204602E-14	10.87333333	5.403333333
85363	'TRIM5'	1.093168919	4.03278354883242E-16	8.273333333	4.023333333
1890	'TYMP'	1.435576721	1.8084277259722798E-40	189.31	73.04666667
9246	'UBE2L6'	1.285037325	5.367429094772281E-44	237.06	101.83
11274	'USP18'	2.097974021	2.16718371948987E-55	27.68666667	7.016666667
9589	'WTAP'	1.03318201	5.367429094772281E-44	217.11	109.99
54739	'XAF1'	1.731518529	8.09306569612812E-75	43.64666667	13.88333333
80149	'ZC3H12A'	1.051227109	8.508694006843921E-31	51.45333333	26.04333333
170689	'ADAMTS15'	-1.298804758	2.27074139246757E-23	2.716666667	6.886666667
11094	'CACFD1'	-1.087245621	3.87448242674885E-8	1.876666667	4.276666667
160364	'CLEC12A'	-1.089744015	8.14726725292676E-12	7.363333333	16.48
81035	'COLEC12'	-1.062972209	8.38909599311781E-17	3.433333333	7.516666667
3579	'CXCR2'	-1.062657375	2.27008331251821E-6	1.65	3.623333333
2167	FABP4'	-1.185423596	3.63990171045858E-8	14.23666667	33.76666667
10223	'GPA33'	-1.76987335	6.0560781681563105E-15	1.136666667	4.06
2857	'GPR34'	-1.016387896	2.67865387764908E-6	2.51	5.336666667
389119	'INKA1'	-1.117790073	5.103222912531E-4	3.003333333	6.793333333
22914	KLRK1'	-1.822223018	1.39894287418304E-18	2.41	8.92
124904248	'LOC124904248'	-1.00067609	2.6585291802783E-5	2.56	5.363333333
91523	'PCED1B'	-1.157796776	6.2542533377929E-7	2.003333333	4.543333333
5166	PDK4'	-1.172974782	9.29061070256188E-7	1.033333333	2.45
79948	'PLPPR3'	-1.002396707	1.5699923893770402E-15	8.583333333	18.04
5657	'PRTN3'	-1.108818014	6.0402417789994405E-21	62.12666667	140.1166667
140738	'TMEM37'	-1.271017332	2.87198576783281E-13	4.186666667	10.56666667
79639	'TMEM53'	-1.014851966	2.38039924275E-4	1.516666667	3.223333333
7277	'TUBA4A'	-1.131660308	2.19813802166126E-5	1.61	3.683333333
9099	'USP2'	-1.116379935	6.852688657054391E-9	1.956666667	4.623333333
11326	VSIG4'	-1.054955353	6.85059118275568E-26	26.43333333	56.51666667

Table S2. DEGs in macrophages infected with pH 9.0 *E. faecalis* MVs vs Control (|Log2 fold change| ≥1)

Gene ID	Gene Symbol	log2 (pH9MVs / Control)	Qvalue (pH9MVs / Control)	Control Average FPKM	pH9MVs Average FPKM
2180	'ACSL1'	1.37668831	2.28973186468151E-64	75.67	182.52
92	'ACVR2A'	1.2947198	5.56705980827987E-35	4.216666667	9.626666667
100	'ADA'	2.044683935	9.80374026056876E-79	23.82666667	91.64666667
27299	'ADAMDEC1'	1.130673569	4.7022434537299496E-57	305.8566667	623.92
133	'ADM'	1.205004355	3.49620080330392E-7	2.993333333	6.433333333
272	'AMPD3'	1.108282321	3.43841763085683E-40	40.77	81.67666667
651746	'ANKRD33B'	1.08476494	4.31919562699185E-14	1.193333333	2.37
339416	'ANKRD45'	1.423310427	3.65963700920869E-16	2.323333333	5.83
80833	'APOL3'	1.452866142	2.76651350155389E-12	2.06	5.206666667
467	'ATF3'	1.214609644	8.148564705425781E-21	8.353333333	17.92333333
22809	'ATF5'	1.041967708	1.6705184398274298E-30	72.93333333	140.1033333
558	'AXL'	1.033772736	5.6727037258272797E-36	28.42	54.06333333
9334	'B4GALT5'	1.017673227	1.5661505825764802E-31	12.25666667	23.05333333
116071	'BATF2'	1.93754027	4.3560006334842896E-18	1.49	5.276666667
597	'BCL2A1'	1.81965904	6.05834433520833E-39	17.73	58.93
602	'BCL3'	2.176347962	4.61542382935034E-65	10.75333333	45.1
283149	'BCL9L'	1.158392073	3.39039782692756E-24	3.5	7.273333333
7439	'BEST1'	1.074631361	7.17584375098724E-17	6.39	11.53
79365	'BHLHE41'	1.363323023	2.12419954293528E-19	2.246666667	5.373333333
330	'BIRC3'	2.768728091	6.9482349259591305E-133	2.786666667	17.24333333
154743	'BMT2'	1.502907422	2.49208922920747E-43	5.143333333	13.56333333
684	'BST2'	1.075167781	1.09208891192453E-15	365.5766667	722.6466667
713	'C1QB'	1.264959283	4.641745492690921E-15	8.563333333	19.24666667
838	'CASP5'	2.115415557	3.76381091254254E-22	2.46	9.94
6347	'CCL2'	4.305209947	0	31.62333333	586.3233333
6364	'CCL20'	3.811448669	3.7471180946025295E-114	7.05	92.83333333
6348	'CCL3'	3.584656696	4.3735160567444405E-145	31.97	360.3433333
6349	'CCL3L1'	4.879017994	3.67942053392708E-89	2.37	63.90666667
414062	'CCL3L3'	4.552707119	4.3111233324239404E-200	9.123333333	199.72
6351	'CCL4'	5.340750299	0	15.50333333	590.1633333
9560	'CCL4L2'	5.978020246	0	8.393333333	495.3
6352	'CCL5'	1.653773661	1.93715482505791E-75	82.58666667	242.36
6355	'CCL8'	5.28596831	8.10822497310728E-167	3.203333333	116.9733333
100133941	'CD24'	1.828475042	1.64544074616664E-40	4.02	13.68
958	'CD40'	1.90863327	3.56153509251487E-65	20.73	72.69333333
962	'CD48'	2.397512825	7.42962314633462E-56	13.31333333	65.62
965	'CD58'	1.179959552	7.47328913834746E-24	39.32333333	83.39
9308	'CD83'	1.856167296	1.28060944307247E-73	12.91333333	43.58
11135	'CDC42EP1'	1.247633722	6.269949595913409E-11	2.943333333	6.513333333
10435	'CDC42EP2'	1.406318506	9.94107668131752E-10	1.696666667	4.193333333
9435	'CHST2'	1.369078184	1.0273735233977801E-45	15.18	36.48333333
50856	'CLEC4A'	1.192621064	3.83001678299205E-12	6.7	14.32
26253	'CLEC4E'	3.180943627	4.16650641334436E-82	2.18	18.43666667
129607	'CMPK2'	2.503158984	3.35095350377485E-88	6.246666667	32.73666667
8506	'CNTNAP1'	1.0882475	1.15794662240177E-13	1.973333333	3.903333333
51232	'CRIM1'	1.877271551	9.59936609705902E-57	7.046666667	23.94333333
1435	'CSF1'	1.053305905	4.67030481324197E-8	1.683333333	3.09
2919	'CXCL1'	3.391045587	2.4255632121660202E-169	14.87333333	145.6566667
3627	'CXCL10'	3.821407393	2.8898815999445797E-187	10.18666667	134.5566667
6387	'CXCL12'	1.318231975	1.5080562653797302E-15	3.363333333	7.566666667
10563	'CXCL13'	4.349000314	4.38357335892939E-70	1.346666667	25.58666667
9547	'CXCL14'	2.410675234	9.09105999793527E-63	4.473333333	22.18
2920	'CXCL2'	3.527354079	1.19552670430337E-110	4.676666667	50.44666667
6372	'CXCL6'	3.2653139	6.51951079322388E-102	3.543333333	31.74333333
3576	'CXCL8'	4.418389112	0	49.42333333	992.3333333
140947	'DCANP1'	1.913046875	2.31376106919755E-64	11.47	40.23
55601	'DDX60'	1.607950356	6.115708158358891E-54	6.403333333	18.16333333
79132	'DHX58'	1.152079332	3.4189523523087304E-17	7.593333333	15.62333333
54567	'DLL4'	2.477607823	8.43280879896225E-101	3.733333333	19.32
126820	'DNAI3'	1.616428144	9.94936891044468E-21	2.203333333	6.283333333
55332	'DRAM1'	1.796260467	1.57134104961148E-79	14.82333333	47.63333333
151636	'DTX3L'	1.131573619	6.83187260535303E-32	16.44333333	33.47
1844	'DUSP2'	1.128810241	3.30864145575643E-6	2.713333333	5.556666667
10148	'EBI3'	2.723840916	7.15676892924898E-143	46.56	287.5966667
10938	'EHD1'	2.047862212	2.77794487885668E-102	9.513333333	36.63333333
5610	'EIF2AK2'	1.312864657	2.58277126389732E-35	13.96	32.16666667
100170841	'EPOP'	1.014660454	5.63687958739646E-12	3.566666667	6.716666667
94240	'EPSTI1'	1.946981046	5.70822105153471E-86	8.913333333	33.77
2247	'FGF2'	1.458407244	1.0457571316729E-19	1.243333333	3.196666667
24147	'FJX1'	1.106823434	6.73348157108601E-8	2.873333333	5.746666667
91010	'FMNL3'	1.619691791	3.52531694464765E-54	2.66	8.3
2495	'FTH1'	1.014394376	1.2410747002006798E-22	5824.833333	11014.21
53826	'FXYD6'	1.114517355	1.50114146517254E-16	12.07333333	24.47666667
8324	'FZD7'	1.003201289	8.64605842521852E-14	2.99	5.573333333
2633	'GBP1'	2.257911327	4.57114539828356E-100	5.403333333	24.07666667

2634	'GBP2'	1.031849468	1.78934115466212E-21	14.35666667	27.38333333
115361	'GBP4'	2.036029059	6.17746507435133E-60	1.726666667	6.566666667
115362	'GBP5'	2.806576241	5.03798320679477E-129	1.446666667	9.403333333
2643	'GCH1'	2.273573334	8.89238687891958E-33	1.366666667	5.693333333
169792	'GLIS3'	1.919133211	5.5277709981488E-48	1.106666667	4.113333333
2766	'GMPR'	1.632342236	2.0451325500025E-7	1.28	3.703333333
29933	'GPR132'	1.305584366	3.70211906957351E-39	9.396666667	21.57666667
353345	'GPR141'	1.081440675	4.30727465673134E-22	9.47	18.57333333
2859	'GPR35'	1.051082832	8.380555877001709E-22	13.11333333	25.41
8111	'GPR68'	1.018305459	3.94723601404981E-10	3.183333333	6.086666667
53831	'GPR84'	1.768473875	2.20740150039248E-34	6.456666667	20.55333333
54329	'GPR85'	1.131742484	1.3977726144694499E-22	8.57	17.59666667
57655	'GRAMD1A'	1.360096825	6.936954752065539E-40	16.57333333	39.36333333
3055	'HCK'	1.100279737	1.51533226859831E-35	115.97	231.97
85441	'HELZ2'	1.342913099	3.5295396413189897E-34	5.873333333	13.88333333
51191	'HERC5'	1.056671091	2.30690562946249E-27	11.71333333	22.67666667
55008	'HERC6'	1.958191984	9.39544070486202E-84	7.973333333	29.07666667
8820	'HESX1'	1.101073835	7.09819729444607E-11	5.926666667	13.21333333
3081	'HGD'	1.295370133	5.98051598244185E-8	2.576666667	5.903333333
3096	'HIVEP1'	1.288704462	1.1008740596340001E-16	1.56	3.53
9955	'HS3ST3A1'	1.425023755	6.20463841428367E-15	1.24	3.046666667
9953	'HS3ST3B1'	2.355077241	8.94590875183509E-49	1.03	4.893333333
3383	'ICAM1'	2.015999898	5.61482537661737E-147	74.75333333	281.5
23308	'ICOSLG'	1.680491898	5.5130820759333E-77	4.33	12.76333333
8870	'IER3'	3.004654386	4.0922520559579204E-91	5.98	44.90333333
51278	'IER5'	1.161843688	1.82132744247133E-36	21.64333333	45.13666667
3429	'IFI27'	2.267336214	2.94768031244786E-50	235.5633333	1064.466667
3430	'IFI35'	1.359853397	2.33170511582221E-29	68.15	163.6566667
10561	'IFI44'	2.158624086	1.33846873699913E-101	32.21333333	134.8133333
10964	'IFI44L'	3.754851137	0	3.336666667	41.83
2537	'IFI6'	2.338098456	3.06598425526753E-76	341.06	1620.04
64135	'IFIH1'	1.360105905	1.42866189276369E-73	29.21666667	69.90333333
3434	'IFIT1'	2.335818519	1.86347185137967E-70	4.526666667	21.32
3433	'IFIT2'	2.345446767	5.579065457128419E-79	2.786666667	13.17
3437	'IFIT3'	2.423450256	1.3592289258096999E-117	8.326666667	41.62333333
24138	'IFIT5'	1.542914805	4.399962435070729E-22	1.653333333	4.48
8519	'IFITM1'	3.294080713	5.95077712134617E-153	119.4233333	1103.853333
10410	'IFITM3'	2.099166152	2.65884524455423E-75	550.4566667	2226.46
3460	'IFNGR2'	1.036854939	9.41408625874703E-39	219.3333333	420.8033333
3486	'IGFBP3'	2.332012925	1.09660103071698E-186	139.5933333	654.7066667
3601	'IL15RA'	2.064866421	4.1650568220922195E-18	1.476666667	5.896666667
8809	'IL18R1'	2.767027026	1.31299554545555E-68	1.243333333	7.77
3553	'IL1B'	3.941232361	0	13.68666667	196.1566667
51561	'IL23A'	1.353207129	9.71110930706406E-12	6.83	16.35
9235	'IL32'	2.952680597	3.21316424963169E-87	19.21	139.75
259307	'IL4I1'	1.874044614	2.92318716232807E-88	98	335.37
3575	'IL7R'	1.830880039	6.91957679973071E-35	1.97	6.523333333
3656	'IRAK2'	3.112512917	3.72019711478383E-104	1.71	13.78666667
3665	'IRF7'	1.588340422	9.16037743534002E-32	14.33	38.13
9636	'ISG15'	2.963667924	7.44707847273554E-18	70.79666667	521.8666667
3669	'ISG20'	1.29774501	2.0256785541837E-8	2.48	5.796666667
3672	'ITGA1'	2.01877217	6.0942914426376204E-58	1.023333333	3.86
8564	'KMO'	1.32335838	6.32590464380378E-32	4.273333333	9.926666667
8942	'KYNU'	1.787144155	2.5017822744571E-98	59.45333333	200.8666667
27074	'LAMP3'	3.670939737	2.1689381514127798E-134	1.156666667	13.68666667
51056	'LAP3'	1.16190207	3.91243661848979E-51	132.6933333	276.8666667
55323	'LARP6'	1.030069678	2.52302262503818E-9	5.403333333	8.41
3959	'LGALS3BP'	1.287186508	8.09273030888755E-29	13.78	31.34333333
3985	'LIMK2'	1.028630042	6.305069258715059E-16	5.21	9.9
102723996	'LOC102723996'	1.775898374	8.87074574295503E-45	5.983333333	21.39666667
122319436	'LOC122319436'	1.43522677	1.56777795581275E-41	3.663333333	9.21
4047	'LSS'	1.100712357	6.92761671737695E-32	18.41333333	36.48333333
4061	'LY6E'	2.44302227	1.64750549201335E-108	131.7733333	670.2566667
4067	'LYN'	1.393682706	2.18232696926384E-85	67.02333333	163.6966667
23764	'MAFF'	1.684598892	1.9240415524111797E-49	8.706666667	26.04666667
84557	'MAP1LC3A'	2.032086303	1.64881349099222E-30	9.183333333	35.34
4082	'MARCKS'	1.435707424	3.1427043019773103E-58	51.56	129.5966667
65108	'MARCKSL1'	1.122419153	4.93284373886842E-35	36.43333333	73.98333333
255231	'MCOLN2'	1.462421007	2.24999474775106E-73	25.11	64.27666667
4223	'MEOX2'	1.813681713	1.81491281011931E-15	1.2	3.936666667
11343	'MGLL'	1.707791278	2.02935474164159E-64	7.633333333	23.12333333
4312	'MMP1'	1.790755336	7.569526598988E-23	2.523333333	8.1
4321	'MMP12'	2.356480791	1.00200194005369E-30	1.63	7.79
64386	'MMP25'	1.492881787	2.16775635342397E-33	4.1	10.54333333
91283	'MSANTD3'	1.028162463	1.2317053861511099E-16	10.91666667	20.96666667
9242	'MSC'	2.523222136	1.55043213112259E-89	9.466666667	50.79333333
4493	'MT1E'	1.022966604	3.956933523421E-4	27.52666667	53.99666667
4502	'MT2A'	1.919676134	8.10467508731775E-28	48.59666667	177.08

4599	'MX1'	2.397869028	6.61175466664522E-181	20.03333333	98.86
4600	'MX2'	2.117165846	3.98694474074846E-159	19.77	79.73666667
10135	'NAMPT'	1.238159951	1.04988139028324E-46	31.38666667	68.93666667
653361	'NCF1'	1.943034949	1.77819441129513E-84	95.33666667	342.68
135112	'NCOA7'	1.028962744	6.24264128597731E-27	21.61666667	43.39
4790	'NFKB1'	1.410149392	2.5192110148159903E-61	21.19	52.58
4791	'NFKB2'	1.458515867	4.5794328379564204E-60	15.68666667	39.98
4792	'NFKBIA'	1.711273125	2.11541626820249E-85	138.92	424.8066667
64332	'NFKBIZ'	1.231924376	1.37115391887953E-38	15.12666667	33.17666667
140688	'NOL4L'	1.451057707	4.23952308666684E-32	3.443333333	8.576666667
10062	'NR1H3'	1.910492696	7.78970029697716E-70	26.75666667	90
8828	'NRP2'	1.227359885	2.2695122682463499E-41	7.063333333	15.44666667
221294	'NT5DC1'	1.28923173	6.187133909265411E-45	9.473333333	22.58333333
4938	'OAS1'	1.469215858	2.25049634994357E-52	61.38666667	160.07
4939	'OAS2'	1.983881319	1.28693491619839E-97	31.92	114.0733333
4940	'OAS3'	2.172146906	1.3997653280217201E-117	24.55333333	103.01
8638	'OASL'	2.243075205	4.7748363271081904E-60	3.1	13.66333333
440836	'ODF3B'	1.180049254	1.21648163421812E-9	8.613333333	18.49333333
5027	'P2RX7'	1.411860734	5.97395256908298E-35	6.323333333	15.84666667
84875	'PARP10'	1.014755754	8.81945957473289E-24	27.66333333	52.1
54625	'PARP14'	1.490326784	1.20406505071499E-68	12.49333333	32.62666667
83666	'PARP9'	1.623354123	3.5292922529572098E-90	22.54666667	64.83333333
80380	'PDCD1LG2'	1.04724836	7.88365362778043E-9	3.343333333	6.433333333
5142	'PDE4B'	1.593287543	1.0554997755892799E-42	4.203333333	11.72666667
9659	'PDE4DIP'	1.469937224	4.4457892568004503E-60	5.906666667	17.50333333
10630	'PDPN'	1.002757663	3.81469376852934E-9	3.936666667	7.36
57162	'PELI1'	1.535199016	1.33912725927642E-46	15.30333333	41.08333333
11040	'PIM2'	1.840455645	1.8574237360801E-82	18.59666667	62.07
11142	'PKIG'	1.984157937	1.92310066373417E-53	10.27	37.21
5341	'PLEK'	1.044801243	2.8629537963906503E-47	186.15	357.4266667
5359	'PLSCR1'	1.412264204	5.274309089164851E-68	70.16333333	174.4633333
5361	'PLXNA1'	1.118554551	2.1037787520822998E-30	7.456666667	15.05333333
285848	'PNPLA1'	1.215559425	1.8025295086294699E-13	2.963333333	6.323333333
87178	'PNPT1'	1.047321637	2.27627638181749E-33	13.98666667	26.68
9050	'PSTPIP2'	1.776221724	9.48617193769872E-66	7.036666667	22.43333333
5724	'PTAFR'	1.818828719	2.40787045507816E-107	11.74666667	38.56333333
5732	'PTGER2'	1.652920339	3.81819607732003E-16	1.663333333	4.866666667
5739	'PTGIR'	2.030551285	1.00668371059297E-29	2.383333333	8.9
5806	'PTX3'	1.917983516	1.43798772827289E-68	10.38	36.55333333
25797	'QPCT'	1.677518142	1.19609914209575E-34	7.176666667	21.39333333
153020	'RASGEF1B'	1.581249083	7.869837418485501E-18	1.673333333	4.676666667
5971	'RELB'	1.391329451	1.30107797343593E-38	20.64333333	50.62
23180	'RFTN1'	1.808374457	3.97802013254124E-88	10.53666667	34.12
23179	'RGL1'	1.457529698	3.12133868054054E-75	19.46	49.68333333
6004	'RGS16'	1.562608662	1.03668123973245E-29	4.603333333	12.68
23433	'RHOQ'	1.236492065	9.20896511671667E-48	33.23666667	72.77
23586	'RIGI'	1.74055789	1.0453495690154099E-70	5.81	18.08666667
390	'RND3'	1.093586922	1.09302224065051E-30	22.41	44.46
255488	'RNF144B'	2.398431913	4.39733862224395E-86	2.916666667	14.19666667
127544	'RNF19B'	1.154331425	3.49295638940027E-39	15.91333333	33.08666667
57674	'RNF213'	1.049842064	2.29732922805156E-28	8.706666667	16.98333333
91543	'RSAD2'	2.242891504	1.78365708574907E-83	3.463333333	15.04666667
64108	'RTP4'	1.049498707	9.520197203782181E-9	6.686666667	12.93333333
54809	'SAMD9'	1.347042107	3.3018319760942896E-36	8.46	19.94666667
219285	'SAMD9L'	1.544533258	5.4690195598065196E-43	8.28	22.48
6303	'SAT1'	1.021218436	3.29686444941242E-18	186.6866667	355.07
64218	'SEMA4A'	1.336724745	9.09105999793527E-63	41.98666667	98.84666667
710	'SERPING1'	1.430718769	1.83952618647942E-12	2.46	6.19
54557	'SGTB'	1.031200138	1.48832653013542E-33	19.38333333	36.61
285590	'SH3PXD2B'	1.31009152	2.25857327238809E-34	3.506666667	7.98
6614	'SIGLEC1'	1.751840395	1.96899158330226E-73	11.13666667	34.85
58516	'SINHCAF'	1.020409746	6.95209283923911E-29	51.71666667	97.82666667
57823	'SLAMF7'	3.230124729	1.62294941563326E-272	13.06666667	111.9666667
9120	'SLC16A6'	1.129589101	1.68595882275911E-6	1.073333333	2.18
11182	'SLC2A6'	1.596531176	1.53500078550219E-63	38.48	106.5766667
23657	'SLC7A11'	1.085702681	7.5627049430134E-21	7.103333333	14.04333333
285195	'SLC9A9'	1.045672496	8.216121448808461E-17	4.35	8.273333333
162394	'SLFN5'	1.122203368	1.28286493259135E-32	5.503333333	11.14666667
8303	'SNN'	1.110998098	6.98036207394927E-40	24.80666667	49.87333333
9021	'SOCS3'	1.928092413	5.48374914258981E-41	4.083333333	14.57666667
6648	'SOD2'	3.04784739	1.97346504361418E-301	112.6933333	771.55
3431	'SP110'	1.095217272	9.276172413533819E-36	24.41	46.38333333
8878	'SQSTM1'	1.138111072	1.91165243592809E-52	218.1433333	447.6133333
26228	'STAP1'	1.681503868	3.6027099186016898E-12	1.503333333	4.713333333
6772	'STAT1'	1.955689037	1.48549228207063E-139	61.06	221.39
9263	'STK17A'	1.313078778	1.1332026331064099E-36	5.93	13.67666667
8676	'STX11'	1.092342883	5.9270385826558E-22	3.983333333	7.853333333
6819	'SULT1C2'	1.832507511	1.2350793523443302E-15	1.04	3.443333333

92610	'TIFA'	1.134705297	1.11320008880288E-22	5.543333333	11.32333333
497189	'TIFAB'	2.092309399	4.0387153841590996E-115	7.7	30.51333333
134285	'TMEM171'	1.740384895	4.40513339792465E-6	1.066666667	3.33
7124	'TNF'	2.810279624	1.1018946340593799E-126	14.13	91.93333333
7127	'TNFAIP2'	2.006069712	6.26826447457789E-100	25.59333333	96.18333333
7128	'TNFAIP3'	1.538507693	1.92346087912514E-67	56.32333333	151.9933333
7130	'TNFAIP6'	4.784511033	0	5.44	139.7733333
25816	'TNFAIP8'	1.126410928	1.18363834217612E-38	28.41333333	57.48
3604	'TNFRSF9'	3.035381964	2.58450728465979E-166	4.636666667	31.77
8743	'TNFSF10'	1.92320583	5.2359159770923205E-61	9.136666667	32.21
10673	'TNFSF13B'	1.710217893	1.82506577918765E-34	5.013333333	15.28333333
10318	'TNIP1'	1.089151438	1.12729330626019E-37	49.01666667	97.63333333
58476	'TP53INP2'	2.29210091	4.9919448717341E-87	3.29	15.04333333
7185	'TRAF1'	3.41263782	3.4157612945852004E-174	2.036666667	20.99666667
10346	'TRIM22'	2.239089271	5.31844451591136E-97	14.33666667	62.87666667
53840	'TRIM34'	1.005330621	7.93666597596914E-12	5.403333333	10.09
85363	'TRIM5'	1.099120696	3.6751154902790505E-15	4.023333333	8.04
1890	'TYMP'	1.411157411	2.5974293229662897E-30	73.04666667	182.06
9246	'UBE2L6'	1.296061064	1.21900217740732E-44	101.83	233.78
7357	'UGCG'	1.184897733	1.03335201406061E-32	15.18333333	31.17666667
219333	'USP12'	1.123730411	7.66436395622184E-26	6.173333333	12.52
11274	'USP18'	2.214864649	1.87592809419061E-74	7.016666667	29.25
7424	'VEGFC'	1.380008232	2.83506890144814E-6	1.076666667	2.613333333
81552	'VOPP1'	1.048729581	1.6090805724193702E-31	40.73333333	78.53
9589	'WTAP'	1.537699831	5.0443272356129196E-80	109.99	301.4666667
54739	'XAF1'	1.729728121	1.66242344927449E-79	13.88333333	42.85333333
65986	'ZBTB10'	1.024763334	4.22774159074662E-17	3.53	6.69
80149	'ZC3H12A'	1.436424358	1.2977553218953298E-56	26.04333333	65.64666667
90874	'ZNF697'	1.008964009	7.21600624192053E-14	3.876666667	7.283333333
170689	'ADAMTS15'	-1.741170136	1.56777795581275E-41	6.886666667	1.926666667
154	ADRB2'	-1.14633207	8.15348899691239E-5	2.796666667	1.18
347902	'AMIGO2'	-1.014537257	2.15149065277255E-7	3.8	1.74
93010	'B3GNT7'	-1.205524739	5.84245188581227E-16	7.383333333	2.983333333
54058	'C21orf58'	-1.147703355	4.32661756042232E-6	3.553333333	1.466666667
11094	'CACFD1'	-1.719645681	1.5909968582703601E-15	4.276666667	1.216666667
160364	'CLEC12A'	-1.235590926	1.81690261479335E-12	16.48	6.55
81035	'COLEC12'	-1.334966028	1.96394040043484E-28	7.516666667	2.77
3579	'CXCR2'	-1.257331384	3.46951584006897E-8	3.623333333	1.42
115265	'DDIT4L'	-1.063701112	3.3385098061103702E-21	20.52666667	9.136666667
64174	'DPEP2'	-1.12799099	7.892634472437E-4	4.08	1.843333333
375704	'ENHO'	-1.385085103	1.94277487078377E-6	9.213333333	3.31
2167	FABP4'	-1.354680041	1.81835347662345E-10	33.76666667	12.41666667
121512	'FGD4'	-1.172373494	1.81491281011931E-15	3.7	1.493333333
10223	'GPA33'	-1.810887992	6.00075644359352E-16	4.06	1.08
2857	'GPR34'	-1.453355137	3.3965457581441694E-11	5.336666667	1.806666667
3034	'HAL'	-1.024903609	1.52137286203671E-15	9.256666667	4.206666667
3101	HK3'	-1.426488423	2.93271978929521E-16	5.05	1.753333333
3399	'ID3'	-1.549733044	7.194539428294E-4	3.546666667	1.133333333
127882475	'IFNAR2-IL10RB'	-1.034206496	4.27171461933047E-5	2.95	1.346666667
389119	'INKA1'	-1.45568301	5.35340456276692E-6	6.793333333	2.36
22914	KLRK1'	-2.105721623	7.569526598988E-23	8.92	1.963333333
124904248	'LOC124904248'	-1.175424281	9.63250501828684E-8	5.363333333	2.223333333
100820829	'MYZAP'	-1.257669569	2.84011822661924E-7	3.343333333	1.3
4900	'NRGN'	-1.1855277	8.840461274389581E-15	28.44333333	11.7
91523	'PCED1B'	-1.371756871	1.43188099843973E-9	4.543333333	1.6
116154	'PHACTR3'	-1.008669423	1.19654982783667E-11	11.68666667	5.37
79948	'PLPPR3'	-1.145771534	4.3221839225789605E-20	18.04	7.576666667
5657	'PRTN3'	-1.140647836	1.44475022169809E-23	140.1166667	59.59333333
26191	'PTPN22'	-1.072690643	9.918412089999331E-14	9.586666667	4.25
28984	'RGCC'	-1.299971907	3.167935059654E-4	5.156666667	1.963333333
6038	'RNASE4'	-1.440024003	8.13757651045868E-8	3.95	1.36
6414	'SELENOP'	-1.073384423	1.26158403346699E-5	4.36	1.93
10509	'SEMA4B'	-1.014529631	3.03831456449604E-7	2.993333333	1.396666667
1468	'SLC25A10'	-1.064476091	6.0909361789553E-9	8.346666667	3.673333333
30061	'SLC40A1'	-1.095024304	1.63298151993941E-7	3.426666667	1.483333333
140738	'TMEM37'	-1.798717514	1.25932918120807E-23	10.56666667	2.84
10098	'TSPAN5'	-1.020164843	6.10829068943823E-6	2.756666667	1.28
9099	'USP2'	-1.328765705	3.28986888024262E-12	4.623333333	1.696666667
1462	'VCAN'	-1.111831916	3.42219976667524E-18	13.81	5.733333333
11326	VSIG4'	-1.139706834	2.7565522691067E-31	56.51666667	24.21
80139	'ZNF703'	-1.309024819	2.81230436832601E-30	14.34	5.386666667

Table S3. DEGs in macrophage infected with pH 9.0 *E. faecalis* MVs vs pH 7.0 *E. faecalis* MVs (|Log2 fold change| ≥0.585)

Gene ID	Gene Symbol	log2 (pH9MVs / pH7MVs)	Qvalue (pH9MVs / pH7MVs)	pH7MVs Average FPKM	pH9MVs Average FPKM
6372	'CXCL6'	1.142229812	3.1187431031745E-26	14.60666667	31.74333333
6364	'CCL20'	0.957106543	1.35736674317973E-18	48.4	92.83333333
2919	'CXCL1'	0.922589706	8.48592051203901E-27	77.98666667	145.6566667
135	'ADORA2A'	0.921685127	7.9355305406752E-7	3.423333333	6.343333333
2921	'CXCL3'	0.910808624	5.37196215535983E-8	9.896666667	18.36
6349	'CCL3L1'	0.894729783	2.3928184908347197E-11	34.79666667	63.90666667
5743	'PTGS2'	0.887472796	3.45991629499525E-6	1.613333333	2.933333333
3576	'CXCL8'	0.865225402	3.91708352761508E-8	552.2466667	992.3333333
6355	'CCL8'	0.839711302	1.1422647870545501E-17	66.19666667	116.9733333
9560	'CCL4L2'	0.837081636	1.9723137034617E-8	278.38	495.3
414062	'CCL3L3'	0.83180559	2.51284256022477E-21	113.4466667	199.72
2920	'CXCL2'	0.812451465	7.899932266465341E-15	29.12	50.44666667
57162	'PELI1'	0.791950428	2.09549960045467E-36	24.14333333	41.08333333
7130	'TNFAIP6'	0.791876438	1.39518719692336E-29	82.01333333	139.7733333
366	'AQP9'	0.784030805	1.84820938339E-4	2.146666667	3.62
3620	'IDO1'	0.772526793	7.09042473344447E-13	16.38666667	27.55333333
6351	'CCL4'	0.769693495	1.05216645640101E-6	349.0233333	590.1633333
3553	'IL1B'	0.758168999	5.53628614851573E-44	117.7	196.1566667
8870	'IER3'	0.749847806	2.0336511817682502E-14	27.09333333	44.90333333
9242	'MSC'	0.743924362	1.8818253054887302E-15	30.81666667	50.79333333
3593	'IL12B'	0.743758984	1.78719128756623E-6	5.473333333	9.006666667
2643	'GCH1'	0.737948627	1.05009803002526E-5	3.423333333	5.693333333
6348	'CCL3'	0.721828082	3.47438012349657E-6	220.8633333	360.3433333
7412	'VCAM1'	0.71060078	4.22968873953093E-7	4.196666667	6.746666667
1236	'CCR7'	0.70968609	2.487179261152E-4	4.54	7.306666667
10125	'RASGRP1'	0.707670688	7.85498353668E-4	1.266666667	2.05
58476	'TP53INP2'	0.681817979	2.31988620866215E-15	9.516666667	15.04333333
9953	'HS3ST3B1'	0.679075799	5.17169966145799E-9	3.116666667	4.893333333
7185	'TRAF1'	0.6767887	5.76747446630655E-14	13.18666667	20.99666667
57823	'SLAMF7'	0.654593937	8.741220063926899E-27	72.32333333	111.9666667
54567	'DLL4'	0.653985634	9.8696644504256E-15	12.48666667	19.32
6648	'SOD2'	0.634948419	1.72939398247114E-41	527.5733333	771.55
9308	'CD83'	0.617700618	8.26182567471127E-16	28.88	43.58
6347	'CCL2'	0.607787121	1.42129981032203E-17	389.0066667	586.3233333
3656	'IRAK2'	0.599294581	1.97315418040727E-8	9.253333333	13.78666667
144717	'PHETA1'	-0.647701408	6.39108441412526E-7	7.55	4.74

Table S4. Sequences of RT-qPCR Primers

Genes		Sequence
FABP4	Forward	5′-TGACAGGAAAGTCAAGAGCACC-3′
	Reverse	5′-TCGTGGAAGTGACGCCTTTC-3′
KLRK1	Forward	5′-TCTAGATCAGGAACTGAGGACAT-3′
	Reverse	5′-CAATGCACAAAGGATTCCTGC-3′
PDK4	Forward	5′-ACCAACGCCTGTGATGGATA-3′
	Reverse	5′-AACCAAAACCAGCCAAAGGAG-3′
HK3	Forward	5′-TGAGATCGAAAGTGACAGCC-3′
	Reverse	5′-TGTAGAGCGTTCCATCCACC-3′
IL1B	Forward	5′-ACCAAACCTCTTCGAGGCAC-3′
	Reverse	5′-TGGCTGCTTCAGACACTTGAG-3′
TNF	Forward	5′-TAGCCCATGTTGTAGCAAACC-3′
	Reverse	5′-GCTCTTGATGGCAGAGAGGA-3′
IFIT2	Forward	5′-CACTGCAACCATGAGTGAGAAC-3′
	Reverse	5′-TAGTTGCCGTAGGCTGCTCT-3′
CXCL6	Forward	5′-TGCGTTGCACTTGTTTACGC-3′
	Reverse	5′-GTTCTTCAGGGAGGCTACCAC-3′
CCL8	Forward	5′-CTTGCCCTCCAAGATGAAGGT-3′
	Reverse	5′-TTGGAATGGAAACTGAATCTGGC-3′
GBP1	Forward	5′-CGCTCTTAAACTTCAGGAACAGG-3′
	Reverse	5′-ACATGCCTTTCGTCGTCTCAT-3′
NOD2	Forward	5′-CACCGTCTGGAATAAGGGTACT-3′
	Reverse	5′-TTCATACTGGCTGACGAAACC-3′
CASP1	Forward	5′-TTTCCGCAAGGTTCGATTTTCA-3′
	Reverse	5′-GGCATCTGCGCTCTACCATC-3′
NFKB1	Forward	5′-GGTGCGGCTCATGTTTACAG-3′
	Reverse	5′-GATGGCGTCTGATACCACGG-3′
GAPDH	Forward	5′-ACAACTTTGGTATCGTGGAAGG-3′
	Reverse	5′-GCCATCACGCCACAGTTTC-3′
